# A Re-Analysis of the Cochrane Library Data: The Dangers of Unobserved Heterogeneity in Meta-Analyses

**DOI:** 10.1371/journal.pone.0069930

**Published:** 2013-07-26

**Authors:** Evangelos Kontopantelis, David A. Springate, David Reeves

**Affiliations:** 1 Centre for Primary Care, NIHR School for Primary Care Research, Institute of Population Health, University of Manchester, Manchester, United Kingdom; 2 Centre for Biostatistics, Institute of Population Health, University of Manchester, Manchester, United Kingdom; 3 Centre for Health Informatics, Institute of Population Health, University of Manchester, Manchester, United Kingdom; University Medical Center Göttingen, Germany

## Abstract

**Background:**

Heterogeneity has a key role in meta-analysis methods and can greatly affect conclusions. However, true levels of heterogeneity are unknown and often researchers assume homogeneity. We aim to: a) investigate the prevalence of unobserved heterogeneity and the validity of the assumption of homogeneity; b) assess the performance of various meta-analysis methods; c) apply the findings to published meta-analyses.

**Methods and Findings:**

We accessed 57,397 meta-analyses, available in the Cochrane Library in August 2012. Using simulated data we assessed the performance of various meta-analysis methods in different scenarios. The prevalence of a zero heterogeneity estimate in the simulated scenarios was compared with that in the Cochrane data, to estimate the degree of unobserved heterogeneity in the latter. We re-analysed all meta-analyses using all methods and assessed the sensitivity of the statistical conclusions. Levels of unobserved heterogeneity in the Cochrane data appeared to be high, especially for small meta-analyses. A bootstrapped version of the DerSimonian-Laird approach performed best in both detecting heterogeneity and in returning more accurate overall effect estimates. Re-analysing all meta-analyses with this new method we found that in cases where heterogeneity had originally been detected but ignored, 17–20% of the statistical conclusions changed. Rates were much lower where the original analysis did not detect heterogeneity or took it into account, between 1% and 3%.

**Conclusions:**

When evidence for heterogeneity is lacking, standard practice is to assume homogeneity and apply a simpler fixed-effect meta-analysis. We find that assuming homogeneity often results in a misleading analysis, since heterogeneity is very likely present but undetected. Our new method represents a small improvement but the problem largely remains, especially for very small meta-analyses. One solution is to test the sensitivity of the meta-analysis conclusions to assumed moderate and large degrees of heterogeneity. Equally, whenever heterogeneity is detected, it should not be ignored.

## Introduction

Meta-analysis (MA), the methodologies of synthesising existing evidence to answer a clinical or other research question, is a relatively young and dynamic area of research. The furore of methodological activity reflects the clinical importance of meta-analysis and its potential to provide conclusive answers, rather than incremental knowledge contributions, much more cheaply than a new large Randomised Clinical Trial (RCT).

The best analysis approach is an Individual Patient Data (IPD) meta-analysis, which requires access to patient level data and considerably more effort (to obtain the datasets mainly). However, with IPD data, clinical and methodological heterogeneity, arguably the biggest concern for meta-analysts, can be addressed through patient-level covariate controlling or subgroup analyses when covariate data are not available across all studies.

When the original data are unavailable, researchers have to combine the evidence in a two stage process, retrieving the relevant summary effects statistics from publications and using a suitable meta-analysis model to calculate an overall effect estimate 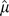. Model selection depends on the estimated heterogeneity, or between-study variance, and its presence usually leads to the adoption of a random-effects (RE) model. The alternative, the fixed-effects model (FE), is used when meta-analysts, for theoretical or practical reasons, decide not to adjust for heterogeneity, or have assumed or estimated the between-study variability to be zero. Different approaches exist for combining individual study results into an overall estimate of effect under the fixed- or random-effects assumptions: inverse variance, Mantel-Haenszel and Peto [1].

Inverse variance approaches are the most flexible and are suitable for continuous or dichotomous data through a fixed-effect or one of numerous random-effects methods. The DerSimonian and Laird [2] method (DL), a moment-based estimator, is the oldest and most widely used random-effects model and justifiably so since it has proven to be remarkably robust in various scenarios [3] including skew-normal and extreme distributions for the effects [4]. Numerous other inverse variance-based approaches to meta-analysis have also been developed: Biggerstaff and Tweedie [5], Follmann and Proschan [6], Sidik and Jonkman [7], maximum likelihood, restricted maximum likelihood and profile likelihood [8]. Most of these are computationally expensive methods and for some convergence to a solution is not guaranteed, but the added complexity does not seem to lead to better overall performance. Although some of the methods perform better on coverage, this is achieved at the expense of power and wider confidence intervals on the effect estimate, especially for meta-analyses of small numbers of studies (with the possible exception of restricted maximum likelihood and profile likelihood) [4], [9], [10].

Mantel-Haenszel [11] methods are suitable for dichotomous data and use a different weighting scheme that depends on the selected effect measure, which can be an odds ratio, a risk ratio or a risk difference. These fixed-effects methods have been shown to have better statistical properties than inverse variance methods when events numbers are low or studies are small [12]. Another fixed-effect alternative is the Peto method which is only suitable for dichotomous outcomes and calculates each study effect as a Peto odds ratio before combining [13]. The method performs well when intervention effects are small or when events are very rare. However, in the presence of heterogeneity, meta-analysts have to revert to inverse variance weighting to use a random-effects model.

For all random-effects models, an accurate estimate of the true between-study variance 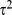 is an important driver of performance. A large 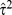 results in wider confidence intervals around the effect estimate with the models taking into account the variability of the effect across studies, while a zero 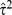 reduces the DL, Sidik-Jonkman, Follmann- Proschan, maximum likelihood and restricted maximum likelihood methods to a fixed-effect model; effectively assuming there is study homogeneity. Although the Biggerstaff-Tweedie and profile likelihood methods take into account the uncertainty in the between-study variance estimate and adjust the confidence intervals accordingly (while all other methods treat the estimate as fixed), their performance is still dependent on an accurate 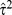.

The between-study variance can be estimated using a number of different approaches. The three mainstream approaches stem from the DL (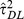), maximum likelihood (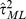) and restricted maximum likelihood (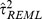) methods and are inherent in them. All the other meta-analysis methods discussed above use 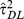, 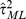 or 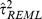 but vary their approach in estimating the effect 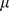. Other methods include the variance component (VC) type estimator [14], [15] and the newer two-step VC and DL [16], model error variance (MV) estimators [17], [18] and Bayes estimators [19].

In practice, 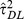 is computed for most meta-analyses and used to quantify heterogeneity in a more interpretable way through Cochran’s 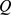
statistic [20] or 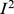 [21]. Although these measures are not without flaws [22], [23], they inform the decision between a fixed-effect and a random-effects meta-analysis model (usually DL). In our experience, researchers are reassured (and reviewers are less critical) when the between study variance estimate is zero, not having to interpret study heterogeneity or deal with model selection dilemmas. Sometimes, researchers opt to use a fixed-effect model even when a small amount of heterogeneity is detected, despite recommendations for the more conservative nature of the random-effects approach and its better performance [3], [4], [23].

The Cochrane Database of Systematic Reviews is the richest resource of meta-analyses in the world with fifty-four active groups responsible for organising, advising on and publishing systematic reviews. Authors are obliged to use RevMan [24], software that has been developed by the Nordic Cochrane Centre and submit the data and analyses file along with the review, contributing to the creation of a vast data resource. Although RevMan offers quite a few fixed-effect choices, only the DerSimonian-Laird random-effects method has been implemented to quantify and account for heterogeneity.

In this paper, following our investigation of effect estimation methods [4], we assess the performance of heterogeneity estimation methods, aiming to bring researchers’ attention to the potential bias from assuming between-study variance is zero. More specifically, we:

1. Compare the performance of between- study variance estimators in various scenarios, through simulations, focusing on non-normal distributions and small numbers of meta-analysed studies. The distributions of the estimates and bias will be compared against the hypothetical distribution used in the simulations. We will also estimate the rates of under- and over-estimation and zero 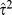, when heterogeneity is present.
2. Investigate the performance of alternative methods, focusing on the 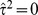 case.
3. Present the distribution of 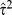 derived from using all meta-analyses in the Cochrane Library (Aug 2012, up to and including CD009888), overall and by method type, as well as details on the number of meta-analysed studies, model selection and zero 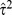. We will also assess the sensitivity of the results and conclusions using alternative models.

## Methods

### Between-study Variance Estimators

Let us consider a group of 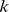 studies with effect size estimates 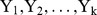 from which we wish to estimate the overall true mean effect 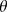. Under the random-effects model, the effect size will vary across studies due to error attributed to within- and between-study variability:
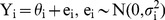
(1)

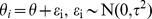
Where 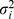 is the within study variance for study 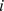 and 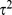 the between-study variance. All variance components are empirically estimated from the available data and the within-study variance estimates 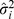 are assumed to equate to their true values, a potentially problematic approach due to the ignored uncertainty [3]. For the between-variance estimate, a plethora of methods exist, frequentist and Bayesian. Again, the estimate uncertainty is almost always ignored and only the point-estimate is incorporated in the random-effects model, while most methods can return a negative estimate and have to be constrained (in which case 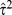 is set to zero). All the methods we assess have been presented in detail by Deeks et al [25], Sidik and Jonkman [18], DerSimonian and Kacker [16] and Rukhin [19] and we are providing a simplified overview. In terms of a-priori assumptions regarding heterogeneity, methods can be categorised in two groups: a) methods which allow heterogeneity to be either zero or positive (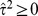); and b) methods that assume heterogeneity is strictly positive, i.e. always greater than zero (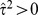). For convenience, we refer to the first group as zero-or-positive (heterogeneity) methods and the second group as positive-only methods.

#### DerSimonian-Laird estimators

The most commonly used approach is the non-iterative method of moments estimator proposed by DerSimonian and Laird [2]:
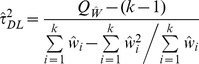
(2)Where 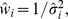 
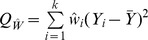
 the Cochran heterogeneity statistic [15] and 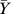 the overall effect estimate using the respective fixed-effects model (i.e. inverse variance, Mantel-Haenszel or Peto).

More recently, DerSimonian and Kacker [16] suggested a two-step approach:
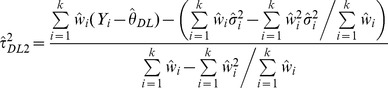
(3)Where 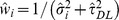 and 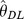 is the effect estimate obtained from the one-step DL method. When equations (2), (3) provide a negative estimate, it is truncated to zero in practice.

We also generated a non-parametric bootstrap [26] version of the DL estimator by randomly sampling studies with replacement 10,000 times and selecting the mean of the truncated estimates. Under this process, for each selected random sample (e.g. for four studies a sample might include studies 1, 1, 2 and 4), between-study variance is estimated using (2) and truncated if negative. The mean of these 10,000 estimates we called 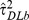.

For a fairer comparison between the DerSimonian-Laird estimator and the Sidik-Jonkman and Rukhin estimators that arbitrarily assume heterogeneity a-priori, we generated estimator 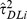 which assumes a between study variance of 0.01 if a zero or negative 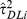 is obtained:
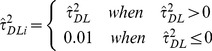
(4)


#### Variance component estimators

Another method of moments estimator was proposed by Hedges [27]:
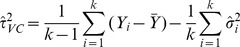
(5)


This simple approach partitions the overall variance estimate into within- and between-variance components and solves for the latter.

A two-step approach has also been suggested [16]:
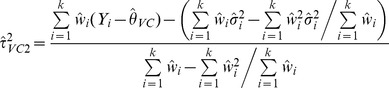
(6)


The estimates from the one-step variance component method are fed into the formula with 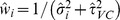 and 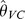 the effect estimate.

In practice, as with the DL estimators, variance components estimators are truncated to zero.

#### Iterative estimators

Maximum-likelihood (ML) is a more computationally demanding iterative approach, assuming within-study variances are known, solves the log likelihood function [8], [18]:
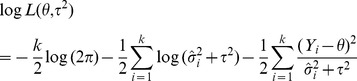
(7)


And the estimate can be obtained from the iteratively solved equation:
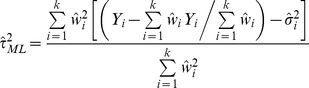
(8)Where 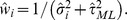


Restricted Maximum Likelihood (REML) estimator is another iterative approach, assuming within-study variances are known, solves the restricted log likelihood function [8], [18]:
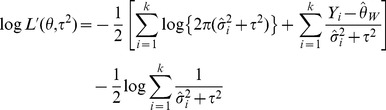
(9)Where 
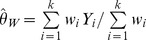
 and 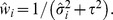


The iterative solution to the equation is the restricted maximum likelihood estimate:
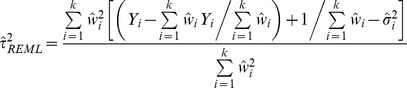
(10)Where 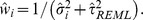


For both ML and REML, truncation to zero at each iteration prevents the estimate from having a negative value. Convergence to a solution is not always successful.

#### Sidik and Jonkman model error variance estimators

Sidik and Jonkman [17] proposed a non-iterative estimator under a different parameterisation of the overall study variability, assuming a positive a-priori value for the between-study variance:
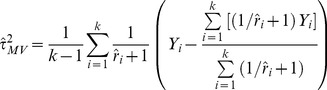
(11)Where 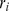 is the within- to between-study variance ratio for study 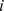 (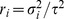). When crude ratio estimates are used as a-priori values, with 
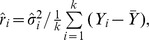
 truncation at zero is not necessary since 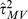 is always positive – we call this estimator 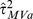. An alternative approach was suggested in which the VC estimator is used to inform on the a-priori values of the ratios, with 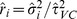. Since the denominator can be zero, an arbitrary value has to be added to it when 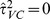. We used 0.01 in compliance with the method’s authors and call this estimator 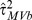. Note that we do not discuss the empirical Bayes estimator proposed by Morris [28], due to its practical similarity to the model error variance estimators [18].

#### Rukhin Bayes estimators

More recently, Rukhin [19] proposed a Bayes estimator that can be reduced to the following when the prior between-study variance is set to zero:
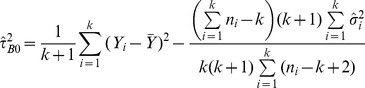
(12)Where 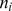 the number of subjects in study 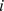. Truncation to zero is necessary since negative estimates are a possibility. Assuming the between-study variance prior is 
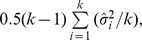
 a simpler and always positive estimator can be obtained:
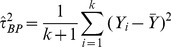
(13)


### Simulations

Our approach was similar to Brockwell’s and Gordon’s [3], generating effect size estimates 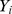 and within study variance estimates 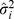 for each simulated meta-analysis study. The distribution of the 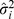 was based on the 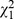 distribution, divided by four and restricted to the (0.009, 0.6) interval, with a mean of 0.173. For the 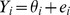, we assumed that 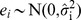. but we simulated various distribution scenarios for 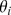 with a mean of 0.5 and variance 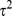 normal skewness  =  0, kurtosis  =  3), moderate skew-normal (skewness  =  1, kurtosis  =  4), extreme skew-normal (skewness  =  2, kurtosis  =  9), uniform, bimodal and double-spike (designed to simulate a situation where study effects take one of two values, a possibility when testing an intervention under two experimental designs). We used three 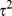 values in an attempt to capture low, medium and large levels of heterogeneity: 0.01, 0.03 and 0.10. These correspond to 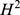 values of 1.18, 1.54 and 2.78 [23] and 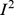 values of approximately, since the measure is not entirely independent of the number of studies in the meta-analysis, 15.1%, 34.9% and 64.1% [21]. To be able to assess 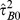 we also independently simulated study sizes 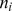 uniformly distributed in the [50,500] range, since estimated study mean 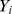 and variance 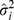 are independent of sample size. For each distributional assumption, 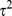. value, and meta-analysis size (which varied from 2 to 30 studies), 10,000 meta-analyses cases were simulated. The full details of the simulation methods have been provided elsewhere [4]. All methods were implemented and analysed in Stata v12.1 for Windows [29].

### Assessment Criteria

The performance of the estimators was assessed using four measures: a) the average bias in the heterogeneity estimate; b) the percentage of zero heterogeneity estimates; c) the coverage probability for the effect estimate; and d) the point and error-interval estimation for the effect.

#### Average bias in the heterogeneity estimate

This is the simplest measure, an aggregate of the bias in the estimate provided by each method (compared to the known 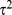 value), across the 10,000 meta-analysis cases in each simulation scenario. This will give us a picture of under-or over-estimation for each method, but not of the overall level of bias (since negative and positive biases will cancel each other out). Therefore, we also calculate and report the average absolute bias.

#### Percentage of zero heterogeneity estimates

We can get a better understanding of a method’s performance by summarising the percentage of cases for which between-study variance is estimated to be zero. Erroneously assuming homogeneity is potentially a big problem for all estimators that do not always return a positive 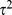.

#### Coverage probability and error-interval estimation for the effect

The accuracy of the between-study variance estimate is just one, albeit very important, parameter for obtaining a precise overall effect estimate, the main goal of all meta-analysis methods. The overall random-effects estimate is usually provided by:
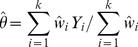
(14)Where 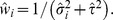 However, the approach is different for the iterative likelihood methods, i.e. maximum, restricted maximum and profile likelihood [8], while variants of (14) have been proposed by Biggerstaff and Tweedie [5] and Sidik and Jonkman [7]. Profile likelihood, which has be shown to be an accurate method [3], [4], uses the 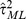 point estimate but also calculates a confidence interval for the estimator and incorporates the uncertainty in the overall effect estimate calculation. The Biggerstaff-Tweedie and Sidik-Jonkman variants provide similar or worse estimates to (14) with 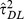 [4] and for convenience we only use the last approach.

The coverage probability is the proportion of 95% confidence intervals for the overall effect estimate that contain the true overall effect 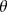, in the 10,000 meta-analysis cases for each simulation scenario. Theoretically, this should be close to 95% but heterogeneity, the number of studies in the meta-analysis and, to a lesser extent, the distribution of the true effects, can affect coverage levels [3], [4].

However, a method can provide high coverage probability rates by overestimating the standard error for the overall effect estimate and thus providing confidence intervals that are overly wide. To better assess the methods we constructed a measure of the accuracy of estimation of the error-interval around the point estimate, computed as the ratio of the estimated confidence interval for the effect, compared to the interval based on the true 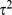:
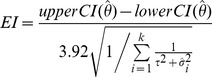
(15)Where 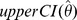 and 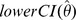 are the upper and lower bounds of the 95% confidence interval for the estimated effect, respectively. The main advantage of this measure is that it allows a straightforward comparison across very different methods [4]. We used the median and the 25^th^ and 75^th^ percentiles of the 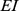 metric, to assess its performance more effectively.

### Cochrane Data

All Cochrane library systematic reviews are constrained to use RevMan [24] and the data for each review, if any relevant studies are identified, are available on the Wiley Group website [30]. Institutional or personal access is required and we developed crawling code in Python [31] to automate the procedure. We were able to access the Wiley website and all Cochrane systematic reviews available in August 2012 (CD000004 to CD009888), downloaded the relevant RevMan files and exported them as *.csv files, before importing in Stata. A total of 3,984 files were available and 3,845 were successfully downloaded (139 files were corrupt). The code has been made available as two supplementary python script files.

Each RevMan file is a systematic review (e.g. olanzapine for schizophrenia). Within a review, various research questions might have been posed (e.g. olanzapine vs placebo, olanzapine vs typical antipsychotics, olanzapine vs atypical antipsychotics) and each question might be investigated across various relevant outcomes, (e.g. global effect: no important clinical response by 6 weeks, adverse events: other). In each of these, an overall meta-analysis is performed on all identified studies, if more than one. However, there might exist a further break-down due to variability in the intervention or the outcome (e.g. drug dosage, or types of other adverse events) and sub-group meta-analyses are then performed. In these cases, an overall meta-analysis which summarises across all studies in the sub-groups can also be executed, if deemed reasonable by the researchers, but this is rarely the case.

From each file, available data for all meta-analyses (e.g. study sizes, event and non-event counts, effect sizes and their variances, alpha level used), overall or subgroup, were collected including the meta-analysis method used and the RevMan output. Method choice in the software is limited to: a) inverse variance fixed-effect or random-effects with DL for continuous or dichotomous outcomes; b) Mantel-Haenszel fixed-effect or Mantel-Haenszel random-effects with DL for dichotomous outcomes; c) Peto fixed-effect for dichotomous outcomes; and d) Peto “O-E” (observed minus expected) fixed-effect time-to-event data. Next, we re-analysed all meta-analyses using the methods implemented in the software [32] and all the random-effects methods described in section 2.1 above, in Stata. This allowed us to identify and report potential bugs in RevMan, assess the sensitivity of the meta-analyses results to the method choice and compare the distributions of the between-study variance estimates obtained by the various methods.

## Results

### Simulations

We present results for just two of the effect-size distributions we simulated, since results for the assessment measures we used were consistent across all distributions: normal and extreme skew-normal. For the iterative methods, non-convergence varied by meta-analysis size and heterogeneity level from 0.1% to 3.5%. Bias assessment is presented in Table S1 in File S1, coverage and zero between-study variance estimate rates in Table S2 and error intervals in Table S3 in File S1.

#### Bias assessment

For low levels of heterogeneity (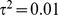; 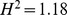; 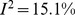), all estimators displayed positive mean bias with ML having the smaller mean and mean absolute bias across all meta-analysis sizes, followed by B0 (for 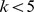) and REML (for 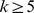). The worst performers were MVa and BP, for 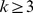 and DLb for 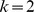. The picture was similar for moderate heterogeneity (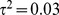; 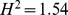; 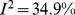) with ML being the best performer and, overall, only slightly underestimating the between-study variance for larger meta-analyses. For considerable heterogeneity (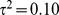; 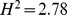; 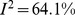), ML still displays the smallest absolute bias levels for all meta-analyses sizes except when 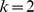, in which case B0 performs slightly better. When it comes to mean bias levels, however, ML consistently underestimates heterogeneity, especially when 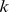 is small. DL and REML seem to be the best performers for large meta-analyses and B0 for very small ones. Levels of DLb bias were the highest for meta-analyses of two studies but acceptable for five or more studies. Across all three heterogeneity levels, observed levels of mean bias and mean absolute bias decreased as the number of studies increased except for the B0 and BP estimators. Interestingly, BP bias was higher for larger meta-analyses (Table S1 in File S1).

#### Coverage and erroneous homogeneity assumption

When heterogeneity was low, DLb, MVb and DLi provided coverage rates close to 95%, while DL, REML and, for larger meta-analyses only, PL also performed well. Amongst the zero-or-positive heterogeneity methods, DLb was the best in terms of detecting any heterogeneity, especially for larger meta-analyses. For moderate heterogeneity levels, DLi and MVb, both of which are positive-only heterogeneity methods, were the best performers overall. In the zero-or-positive estimator group, DLb performed the best in terms of heterogeneity detection while providing good coverage and PL was the best in coverage. For large heterogeneity, MVa had the most accurate coverage levels overall, with BP performing well only for small meta-analyses. Methods DLb and PL also performed well, with the former outperforming all other zero-or-positive methods and the latter providing the best coverage levels. The higher heterogeneity detection rates for DLb are not surprising considering its high bias for small meta-analyses. However, for larger meta-analyses of 5 or more studies, when DLb bias is comparable or even lower to bias observed for other 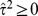 methods, DLb is still the best performer in heterogeneity detection. Note that across all methods, levels of coverage decreased as heterogeneity increased (Table S2 in File S1).

#### Error interval estimation

For low levels of heterogeneity, the positive-only heterogeneity methods DLi and MVb did particularly well, since the assumed minimum degree of positive heterogeneity matched the mean value in the simulation. Amongst the zero-or-positive heterogeneity methods, DLb was the best overall performer in terms of median performance, only slightly over-estimating the error interval for larger meta-analysis. Other good performers were DL, REML and B0 (especially for small study numbers), while MVa, BP and PL provided error intervals that were too wide (PL for smaller and MVa, BP for larger meta-analyses). In terms of the variability in the estimate, B0 was more consistent in providing an accurate estimate for 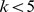 and DL and REML for 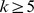. When heterogeneity was moderate, the picture did not change much with the only noticeable differences being the slight improvement in the DLb median estimate and the small performance deterioration for DLi and MVb (since the a-priori assumption is now an under-estimate). For large level of heterogeneity, most methods underestimated the error interval by more than 10% when 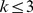. For very small meta-analyses, the positive-only methods BP and MVa methods performed well. DLb was the best overall performer and scored particularly well when 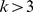 (Table S3 in File S1).

### Cochrane Data

**Figure 1:**
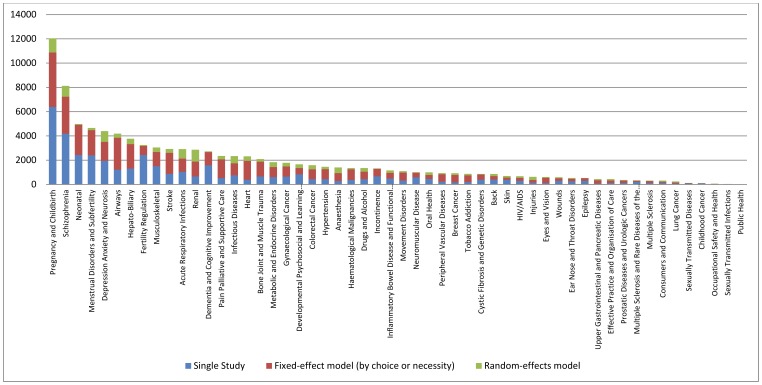
All meta-analyses, including single-study and subgroup meta-analyses.

**Figure 2:**
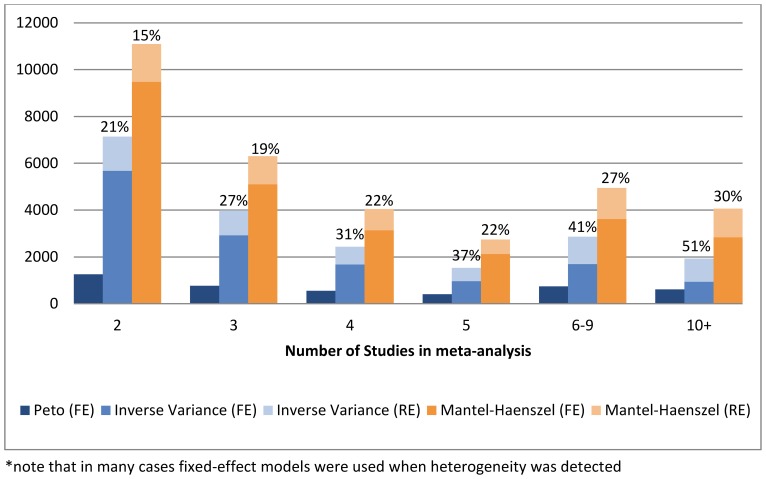
Model selection by number of available studies (and % of random-effects meta-analyses)*.

Of the 3,845 downloaded files (systematic reviews) only 2,801 had identified relevant studies and contained any data. A total of 98,615 analyses were extracted, 57,397 of which were meta-analyses (i.e. combined results across more than one study). Of these, 32,005 were overall meta-analyses and 25,392 were subgroup meta-analyses. Figure 1 provides the analyses counts by Cochrane Group. In these meta-analyses, the estimation of an overall effect was obtained with a Peto method in 4,340 (7.6%) cases, a Mantel-Haenszel method in 33,184 (57.8%) cases and an inverse variance method in 19,873 (34.6%) cases. For the Peto meta-analyses, 585 of these used the “O-E” method. Choice of fixed- or a random-effects model varied by meta-analysis size and method; random-effects models were more prevalent in inverse variance methods and larger meta-analyses (Figure 2).

**Figure 3:**
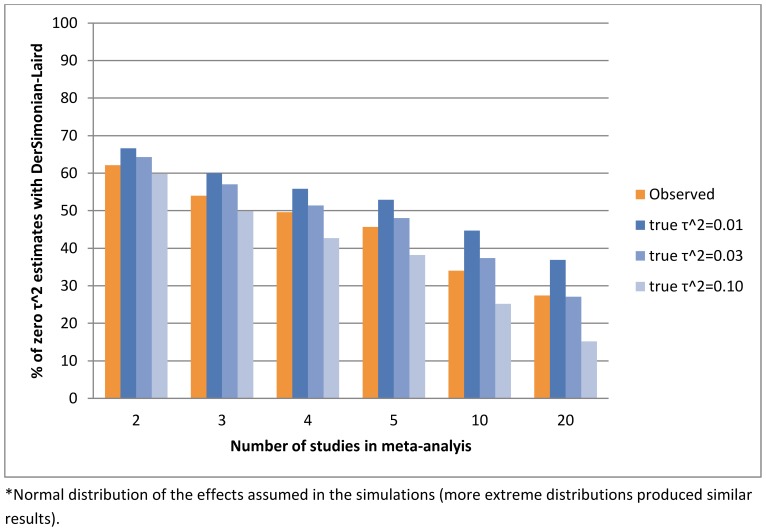
Comparison of zero between-study variance estimates rates in the Cochrane library data and in simulations, using the DerSimonian-Laird method*.

To assess the validity of a homogeneity assumption and, thus, a fixed-effect model choice in Cochrane data meta-analyses, we compared the percentage of zero between-study variance estimates under the DerSimonian-Laird method, in the real and simulated data (Figure 3). To proceed with the comparison we calculated the between-study variance estimate for all Cochrane meta-analyses, since the measure is not reported in RevMan when a fixed-effect method is used. We observed that the percentage of zero between-study variance estimates was lower in the real data than in the low and moderate heterogeneity simulated data. This suggests that the mean true between-study variance is higher than generally assumed but fails to be detected, especially when the number of studies meta-analyses is small.

#### RevMan issues

For the Mantel-Haenszel methods, we encountered some problems, when events or non-events were very rare. When study event or non-event cells were empty, calculations for risk ratios and risk differences (in RevMan v5.2.3) were not in complete agreement with the documentation [32], potentially affecting overall effect estimates. The issues were reported to the Nordic Cochrane Centre.

Prior to version 5.0.17 (1 Dec 2008) the Peto method was not using the correct study (Peto) odds ratio estimates and we encountered a few meta-analyses that had not been updated with the corrections.

#### Method comparison

We applied the methods described in section 2.1 to all 57,397 meta-analyses to assess the distributions of 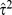 and the sensitivity of the results and conclusions. All comparisons are presented in Tables 1, 2 and 3 but for simplicity we only discuss differences between the standard methods and the bootstrapped DerSimonian Laird (not a perfect method, but one that performs well overall, despite its larger heterogeneity bias for small meta-analyses).

**Table 1:** Variation in terms of statistical conclusion for inverse variance meta-analyses.

		RevMan method					
		Fixed-effect ( 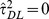 )		Fixed-effect ( 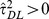 )		Random-effects DL ( 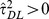 )	
		Counts (cell percentages)		Counts (cell percentages)		Counts (cell percentages)	
Stata method		NS	Sig	NS	Sig	NS	Sig
FE	NS	4494(50.3%)*	0(0.0%)†	1743(35.3%)	0(0.0%)	1622(27.0%)	17(0.3%)
	Sig	0(0.0%)‡	4438(49.7%)§	0(0.0%)	3194(64.7%)	1161(19.3%)	3204(53.4%)
DL	NS	4494(50.3%)	0(0.0%)	1737(35.2%)	831(16.8%)	2783(46.4%)	0(0.0%)
	Sig	0(0.0%)	4438(49.7%)	6(0.1%)	2363(47.9%)	0(0.0%)	3221(53.6%)
DLb	NS	4494(50.3%)	84(0.9%)	1737(35.2%)	936(19.0%)	2777(46.3%)	140(2.3%)
	Sig	0(0.0%)	4354(48.7%)	6(0.1%)	2258(45.7%)	6(0.1%)	3081(51.3%)
VC	NS	4494(50.3%)	38(0.4%)	1739(35.2%)	857(17.4%)	2646(44.1%)	207(3.4%)
	Sig	0(0.0%)	4400(49.3%)	4(0.1%)	2337(47.3%)	137(2.3%)	3014(50.2%)
MVa	NS	4415(49.4%)	309(3.5%)	1738(35.2%)	1021(20.7%)	2733(45.5%)	277(4.6%)
	Sig	0(0.0%)	4069(45.6%)	5(0.1%)	2173(44.0%)	50(0.8%)	2944(49.0%)
MVb	NS	4494(50.3%)	117(1.3%)	1739(35.2%)	878(17.8%)	2680(44.6%)	178(3.0%)
	Sig	0(0.0%)	4321(48.4%)	4(0.1%)	2316(46.9%)	103(1.7%)	3043(50.7%)
BP	NS	4494(50.3%)	532(6.0%)	1737(35.2%)	952(19.3%)	2579(43.0%)	303(5.0%)
	Sig	0(0.0%)	3906(43.7%)	6(0.1%)	2242(45.4%)	204(3.4%)	2918(48.6%)
B0	NS	4494(50.3%)	31(0.3%)	1736(35.2%)	601(12.2%)	2381(39.7%)	120(2.0%)
	Sig	0(0.0%)	4407(49.3%)	7(0.1%)	2593(52.5%)	402(6.7%)	3101(51.6%)
VC2	NS	4494(50.3%)	13(0.1%)	1737(35.2%)	898(18.2%)	2754(45.9%)	167(2.8%)
	Sig	0(0.0%)	4425(49.5%)	6(0.1%)	2296(46.5%)	29(0.5%)	3054(50.9%)
DL2	NS	4494(50.3%)	0(0.0%)	1736(35.2%)	844(17.1%)	2717(45.3%)	117(1.9%)
	Sig	0(0.0%)	4438(49.7%)	7(0.1%)	2350(47.6%)	66(1.1%)	3104(51.7%)
ML	NS	4468(50.0%)	1(0.0%)	1725(34.9%)	442(9.0%)	2272(37.8%)	44(0.7%)
	Sig	0(0.0%)	4404(49.3%)	8(0.2%)	2721(55.1%)	499(8.3%)	3141(52.3%)
	No conv||	26(0.3%)	33(0.4%)	10(0.2%)	31(0.6%)	12(0.2%)	36(0.6%)
REML	NS	4365(48.9%)	14(0.2%)	1730(35.0%)	852(17.3%)	2713(45.2%)	108(1.8%)
	Sig	0(0.0%)	4314(48.3%)	6(0.1%)	2332(47.2%)	66(1.1%)	3103(51.7%)
	No conv||	129(1.4%)	110(1.2%)	7(0.1%)	10(0.2%)	4(0.1%)	10(0.2%)
PL	NS	4440(49.7%)	514(5.8%)	1716(34.8%)	1006(20.4%)	2709(45.1%)	300(5.0%)
	Sig	1(0.0%)	3862(43.2%)	4(0.1%)	2133(43.2%)	50(0.8%)	2881(48.0%)
	No conv||	53(0.6%)	62(0.7%)	23(0.5%)	55(1.1%)	24(0.4%)	40(0.7%)

* Agreement in statistical conclusion (non-significant effect) between RevMan and Stata methods.

† Non agreement in statistical conclusion between RevMan (significant effect) and Stata methods (non-significant effect).

‡ Non agreement in statistical conclusion between RevMan (significant effect) and Stata methods (non-significant effect).

§ Agreement in statistical conclusion (significant effect) between RevMan and Stata methods.

|| Iterative method failed to convergence to a solution.

**Table 2:** Variation in terms of statistical conclusion for Mantel-Haenszel meta-analyses*.

		RevMan method
		Fixed-effect ( 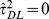 )		Fixed-effect ( 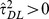 )		Random-effects DL ( 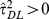 )	
		Counts (cell percentages)		Counts (cell percentages)		Counts (cell percentages)	
Stata method		NS	Sig	NS	Sig	NS	Sig
FE (MH fixed)	NS	8929(70.8%)	0(0.0%)	4311(51.7%)	2(0.0%)	6422(52.5%)	51(0.4%)
	Sig	11(0.1%)	3678(29.1%)	8(0.1%)	3983(47.7%)	1666(13.6%)	4067(33.3%)
	No comp†	0(0.0%)	0(0.0%)	37(0.4%)	3(0.0%)	16(0.1%)	0(0.0%)
DL (MH random)	NS	8882(70.4%)	196(1.6%)	4304(51.6%)	1539(18.4%)	8033(65.7%)	33(0.3%)
	Sig	58(0.5%)	3482(27.6%)	15(0.2%)	2446(29.3%)	55(0.5%)	4085(33.4%)
	No comp†	0(0.0%)	0(0.0%)	37(0.4%)	3(0.0%)	16(0.1%)	0(0.0%)
DLb	NS	8890(70.5%)	336(2.7%)	4306(51.6%)	1676(20.1%)	8023(65.6%)	265(2.2%)
	Sig	50(0.4%)	3342(26.5%)	13(0.2%)	2309(27.7%)	65(0.5%)	3853(31.5%)
	No comp†	0(0.0%)	0(0.0%)	37(0.4%)	3(0.0%)	16(0.1%)	0(0.0%)
VC	NS	8889(70.4%)	282(2.2%)	4298(51.5%)	1601(19.2%)	7844(64.2%)	316(2.6%)
	Sig	51(0.4%)	3396(26.9%)	21(0.3%)	2384(28.6%)	244(2.0%)	3802(31.1%)
	No comp†	0(0.0%)	0(0.0%)	37(0.4%)	3(0.0%)	16(0.1%)	0(0.0%)
MVa‡	NS	8863(70.2%)	787(6.2%)	4303(51.6%)	1882(22.6%)	7982(65.3%)	683(5.6%)
	Sig	19(0.2%)	2876(22.8%)	16(0.2%)	2103(25.2%)	84(0.7%)	3433(28.1%)
	No comp†	58(0.5%)	15(0.1%)	37(0.4%)	3(0.0%)	38(0.3%)	2(0.0%)
MVb	NS	8917(70.7%)	399(3.2%)	4303(51.6%)	1603(19.2%)	7912(64.7%)	374(3.1%)
	Sig	23(0.2%)	3279(26.0%)	16(0.2%)	2382(28.5%)	176(1.4%)	3744(30.6%)
	No comp†	0(0.0%)	0(0.0%)	37(0.4%)	3(0.0%)	16(0.1%)	0(0.0%)
BP	NS	8933(70.8%)	1111(8.8%)	4305(51.6%)	1944(23.3%)	7908(64.7%)	964(7.9%)
	Sig	7(0.1%)	2567(20.3%)	14(0.2%)	2041(24.5%)	180(1.5%)	3154(25.8%)
	No comp†	0(0.0%)	0(0.0%)	37(0.4%)	3(0.0%)	16(0.1%)	0(0.0%)
B0	NS	8887(70.4%)	267(2.1%)	4301(51.5%)	1290(15.5%)	7624(62.4%)	217(1.8%)
	Sig	53(0.4%)	3411(27.0%)	18(0.2%)	2695(32.3%)	464(3.8%)	3901(31.9%)
	No comp†	0(0.0%)	0(0.0%)	37(0.4%)	3(0.0%)	16(0.1%)	0(0.0%)
VC2	NS	8887(70.4%)	222(1.8%)	4301(51.5%)	1622(19.4%)	7978(65.3%)	202(1.7%)
	Sig	53(0.4%)	3456(27.4%)	18(0.2%)	2363(28.3%)	110(0.9%)	3916(32.0%)
	No comp†	0(0.0%)	0(0.0%)	37(0.4%)	3(0.0%)	16(0.1%)	0(0.0%)
DL2	NS	8884(70.4%)	196(1.6%)	4299(51.5%)	1504(18.0%)	7922(64.8%)	116(0.9%)
	Sig	56(0.4%)	3482(27.6%)	20(0.2%)	2481(29.7%)	166(1.4%)	4002(32.7%)
	No comp†	0(0.0%)	0(0.0%)	37(0.4%)	3(0.0%)	16(0.1%)	0(0.0%)
ML	NS	8831(70.0%)	63(0.5%)	4204(50.4%)	679(8.1%)	7113(58.2%)	64(0.5%)
	Sig	42(0.3%)	3571(28.3%)	70(0.8%)	3241(38.8%)	894(7.3%)	3985(32.6%)
	No conv/comp†	67(0.5%)	44(0.3%)	82(1.0%)	68(0.8%)	97(0.8%)	69(0.6%)
REML	NS	8629(68.4%)	212(1.7%)	4271(51.2%)	1423(17.1%)	7792(63.8%)	98(0.8%)
	Sig	56(0.4%)	3374(26.7%)	26(0.3%)	2538(30.4%)	189(1.5%)	3954(32.4%)
	No conv/comp†	255(2.0%)	92(0.7%)	59(0.7%)	27(0.3%)	123(1.0%)	66(0.5%)
PL	NS	8803(69.8%)	846(6.7%)	4260(51.1%)	1679(20.1%)	7876(64.4%)	576(4.7%)
	Sig	15(0.1%)	2766(21.9%)	14(0.2%)	2238(26.8%)	114(0.9%)	3463(28.3%)
	No conv/comp†	122(1.0%)	66(0.5%)	82(1.0%)	71(0.9%)	114(0.9%)	79(0.6%)

* Following the RevMan documentation we did not compute effects for some very rare events or non-events studies (although RevMan v5.2.3 does calculate effects for them) and hence some meta-analyses were not computable; i.e. when the remaining number of eligible studies was one or zero.

† Not computable (see above) or iterative method failed to convergence to a solution.

‡ MVa fails to produce an estimate when all effects are equal, a scenario not unheard of when meta-analysing dichotomous data.

**Table 3:** Variation in terms of statistical conclusion for Peto fixed-effect meta-analyses.

		RevMan method			
		Fixed-effect ( 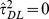 )		Fixed-effect ( 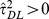 )	
		Counts (cell percentages)		Counts (cell percentages)	
Stata method		NS	Sig	NS	Sig
FE (Peto)	NS	1367(62.0%)	0(0.0%)	1184(55.5%)	0(0.0%)
	Sig	4(0.2%)	835(37.9%)	0(0.0%)	950(44.5%)
DL	NS	1367(62.0%)	0(0.0%)	1178(55.2%)	318(14.9%)
	Sig	4(0.2%)	835(37.9%)	6(0.3%)	632(29.6%)
DLb	NS	1367(62.0%)	36(1.6%)	1177(55.2%)	359(16.8%)
	Sig	4(0.2%)	799(36.2%)	7(0.3%)	591(27.7%)
VC	NS	1367(62.0%)	17(0.8%)	1181(55.3%)	343(16.1%)
	Sig	4(0.2%)	818(37.1%)	3(0.1%)	607(28.4%)
MVa	NS	1366(61.9%)	117(5.3%)	1178(55.2%)	448(21.0%)
	Sig	4(0.2%)	718(32.5%)	6(0.3%)	502(23.5%)
	No comp*	1(0.0%)	0 (0.0%)	0(0.0%)	0 (0.0%)
MVb	NS	1367(62.0%)	39(1.8%)	1180(55.3%)	344(16.1%)
	Sig	4(0.2%)	796(36.1%)	4(0.2%)	606(28.4%)
BP	NS	1369(62.1%)	179(8.1%)	1178(55.2%)	462(21.6%)
	Sig	2(0.1%)	656(29.7%)	6(0.3%)	488(22.9%)
B0	NS	1367(62.0%)	13(0.6%)	1182(55.4%)	266(12.5%)
	Sig	4(0.2%)	822(37.3%)	2(0.1%)	684(32.1%)
VC2	NS	1367(62.0%)	4(0.2%)	1177(55.2%)	362(17.0%)
	Sig	4(0.2%)	831(37.7%)	7(0.3%)	588(27.6%)
DL2	NS	1367(62.0%)	0(0.0%)	1180(55.3%)	333(15.6%)
	Sig	4(0.2%)	835(37.9%)	4(0.2%)	617(28.9%)
ML	NS	1362(61.7%)	1(0.0%)	1174(55.0%)	156(7.3%)
	Sig	4(0.2%)	826(37.4%)	2(0.1%)	790(37.0%)
	No conv†	5(0.2%)	8(0.4%)	8(0.4%)	4(0.2%)
REML	NS	1343(60.9%)	3(0.1%)	1177(55.2%)	293(13.7%)
	Sig	4(0.2%)	819(37.1%)	6(0.3%)	653(30.6%)
	No conv†	24(1.1%)	13(0.6%)	1(0.0%)	4(0.2%)
PL	NS	1364(61.8%)	135(6.1%)	1174(55.0%)	382(17.9%)
	Sig	2(0.1%)	690(31.3%)	1(0.0%)	564(26.4%)
	No conv†	5(0.2%)	10(0.5%)	9(0.4%)	4(0.2%)

* MVa fails to produce an estimate when all effects are equal, a scenario not unheard of when meta-analysing dichotomous data.

† Iterative method failed to convergence to a solution.

**Figure 4:**
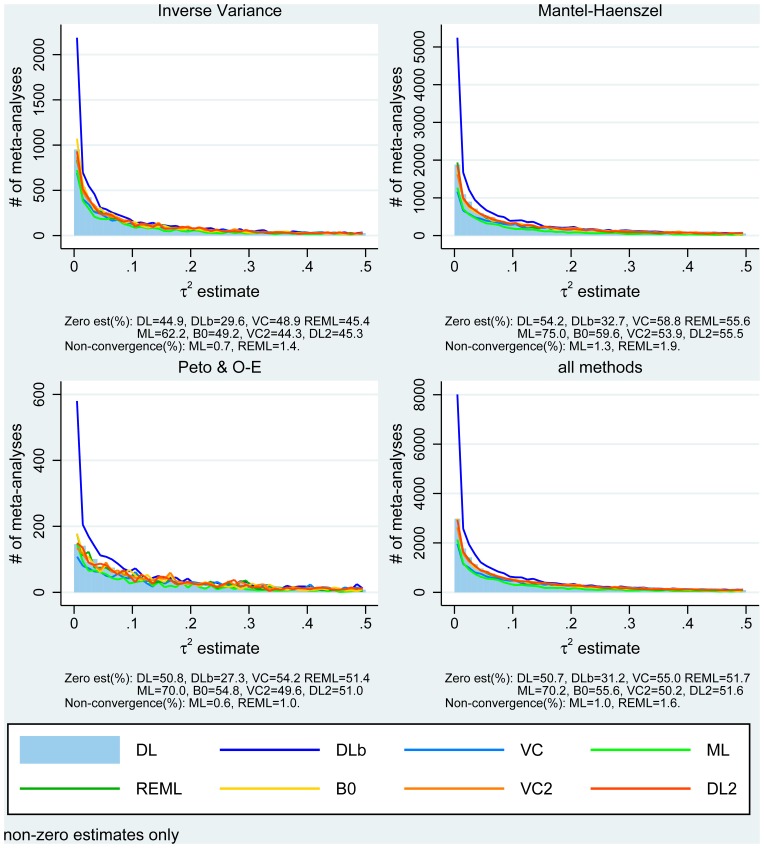
Distribution of between-study variance estimates by method type (including main and subgroup meta-analyses and truncated to 0.5 for better visualisation).

**Figure 5:**
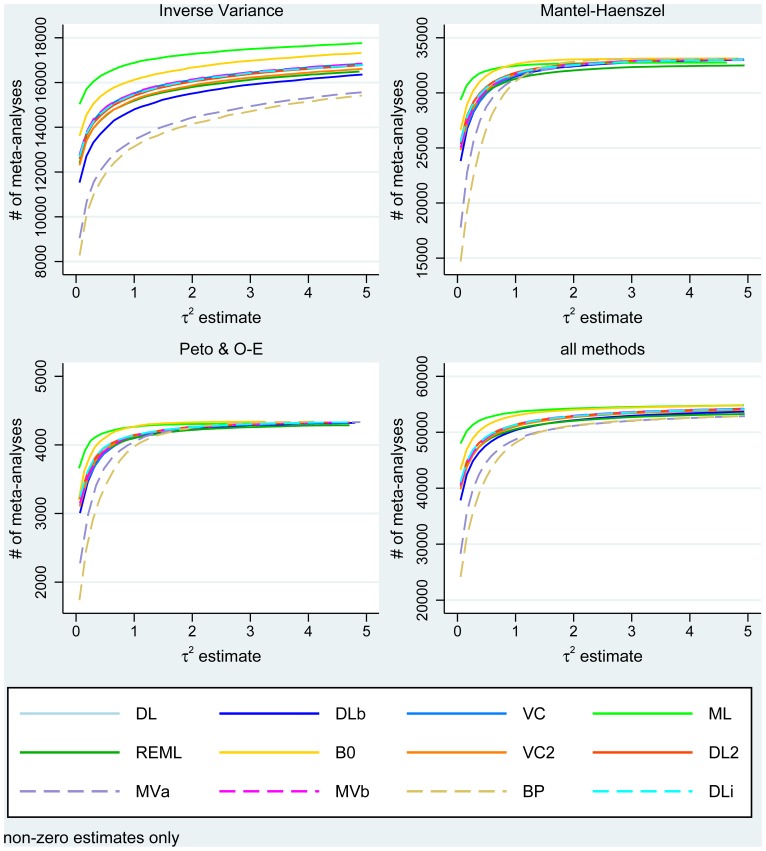
Cumulative distribution of between-study variance estimates by method type (including main and subgroup meta-analyses and truncated to 5 for better visualisation).

The distributions of the estimators that do not assume always present heterogeneity are provided in Figure 4 and cumulative distributions for all methods in Figure 5 (by approach type and overall). For inverse variance and Mantel-Haenszel methods, they are similar and in agreement with the hypothesised 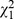 distribution we used for the simulations. For Peto methods, the observed distributions are flatter although the much smaller number of studies might be a factor. As in the simulations, the bootstrapped DerSimonian-Laird method identifies more heterogeneous meta-analyses: overall, 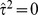 for 31.2% of analyses with DLb and 50.7% with the standard DL. This ‘success’ is partly attributed to the higher positive bias rates for very small meta-analyses. The findings are similar when only focusing on the 32,005 ‘overall’ meta-analyses (Figure S1 in File S1). The better performance of the bootstrap approach in identifying heterogeneity, compared to the standard approach, is consistent across various meta-analysis sizes (Figure S2 in File S1) but more profound when the number of studies is small (Figure S3 in File S1).

For inverse variance methods, the sensitivity of the results to the method choice, in terms of the statistical conclusion, is summarised in Table 1. When 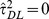, the fixed-effect method is necessarily used in RevMan and, as expected, the conclusions agree completely with what we obtained in Stata when using the same method (DL is reduced to fixed-effect in the absence of heterogeneity). However, the better at detecting heterogeneity DLb method identified heterogeneity for some of these studies, leading to the use of the respective random-effects model and a change in the conclusion to non-significant effect for 84 meta-analyses, approximately 0.9% of the analyses in the group. When 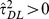, the authors are given a choice between a fixed-effect and a DL random-effects model. Ignoring heterogeneity and proceeding with the former is not prudent, if the authors wish to their findings to be generalizable. In that scenario we identified 6 statistically non-significant effect analyses (0.1%) and 936 significant effect analyses (19.0%) for which the conclusions changed with DLb. When authors more appropriately used the DL random-effects approach, our calculations in Stata agree for the method but with DLb we also identified 6 non-significant effect analyses (0.1%) and 140 significant effect analyses (2.3%) for which the conclusions changed.

For Mantel-Haenszel approaches, we summarise the sensitivity of the conclusions to the method choice in Table 2. When 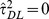, the MH fixed-effect method is used in RevMan and as before, the DLb method identified heterogeneity for some of these analyses and the respective random-effects model produced overall effects for which the conclusion changed from non-significant in 50 cases (0.4%) and from significant in 336 cases (2.7%). When 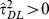 and heterogeneity is ignored through the MH fixed-effect approach, we identified 13 statistically non-significant effect analyses (0.2%) and 1,676 significant effect analyses (20.1%) for which the results changed with DLb. When authors took heterogeneity into account with a DL random-effects approach, we identified 65 non-significant effect analyses (0.5%) and 265 significant effect analyses (2.2%) for which the conclusions changed. The computational issues for the non-iterative methods and some of the changes in statistical conclusions are due to the very rare events (or non-events) disagreement with RevMan.

Table 3 summarises the differences in the statistical conclusions across methods when a Peto fixed-effect approach has been used in RevMan, irrespective of the estimated heterogeneity. When 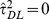, the DLb method identified heterogeneity for some of these analyses and the respective random-effects model produced overall effects for which the conclusion changed from non-significant in 4 cases (0.2%) and from significant in 36 cases (1.6%). When 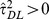, DLb identified 7 (0.3%) and 359 (16.8%) analyses for which the statistical conclusion changed from non-significant and significant respectively. A very few of the changes in the statistical conclusions are attributed to the unapplied Peto odds-ratio corrections in analyses executed in RevMan versions prior to 5.017.

The differences in the statistical conclusions between the standard methods and the DerSimonian-Laird bootstrap, by meta-analysis size, are presented in Tables S4 to S6 in File S1. For inverse variance approaches, differences are more common for smaller meta-analyses and the methods agree more often in meta-analyses of 10 or more studies. For the Mantel-Haenszel and Peto approaches, differences are more consistent across meta-analysis sizes.

## Discussion

The importance of meta-analysis in medical research and its great influence in clinical practice are unquestionable. However, researchers and clinicians alike need to be aware of the potential issues with the current methods and, in particular, the likely fallacy of a homogeneity assumption. A zero between-study variance estimate, which is often observed when the number of studies to be meta-analyses is small, should be a cause for concern and further investigation.

### Strengths and Limitations

The study used a vast resource to meet its goals, all available Cochrane Database of Systematic Reviews data in August 2012, a total of 57,397 meta-analyses. Nevertheless, there are a few limitations that need to be highlighted. Firstly, although the Cochrane library is the biggest resource for systematic reviews and meta-analysis it is not the only one. However, we have no reason to believe that our findings would not be applicable to other resources. Secondly, we used all reported meta-analyses, including 25,392 subgroup meta-analyses, and therefore there are instances where the same study is used in numerous analyses. Although this could potentially affect the distributions of the heterogeneity estimates (but this does not seem to be the case) we felt that it was more important to be inclusive and assess the sensitivity of all meta-analysis, especially since some overall analyses are not performed and only the subgroup ones are reported. Thirdly, for dichotomous outcomes, we did not compare across inverse variance, Mantel-Haenszel and Peto methods, but re-analysed using the same approach for simplicity. Fourth, the true heterogeneity distribution will very likely vary by outcome type or other study characteristics [33] but looking into these sub-categories is beyond the scope of this study.

### Findings

In simulated data with specified degrees of non-zero heterogeneity, we demonstrated that most methods that allow the heterogeneity estimator to be zero, including the common DerSimonian-Laird approach, perform well on average, even for extreme distributions of the effects. However, these methods quite often fail to detect heterogeneity and thus produce biased estimates and conclusions, especially for small meta-analyses. The best method, overall, in particular in terms of detecting heterogeneity when it is present, appeared to be a non-parametric bootstrap of the DerSimonian-Laird, which we have implemented in Stata [34]. Although the method often produces positively biased heterogeneity estimates, especially for small meta-analyses, it performs very well on coverage, error-interval estimation and heterogeneity detection.

Not surprisingly, positive-only heterogeneity methods had high levels of bias which led to error intervals that were very wide, especially for larger meta-analyses. The best balance across all three measures of assessment was achieved by Sidik and Jonkman’s MVb [17]. However, by arbitrarily assuming a minimum level of 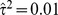 with the standard DerSimonian-Laird model, the same level MVb uses as a starting point, we obtained better results compared to MVb.

Using the Cochrane Database of Systematic Reviews we gained access to data for thousands of meta-analysis and using that resource we were able to further compare the performance of difference meta-analysis methods. The distribution of the between-study variance estimates was found to be consistent across inverse variance, Mantel-Haenszel and Peto approaches and similar to a 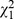 distribution as hypothesised in previous work [3].

A comparison between real and simulated data using the DerSimonian-Laird method suggested that mean true heterogeneity is higher than assumed or estimated in practice (higher than moderate) but the standard method fails to detect it, especially for meta-analyses of few studies. Thus we observed that random-effects methods are much more common in larger meta-analyses. This finding agrees with the conclusion of Turner et al, who found that meta-analysis size had a small effect on heterogeneity [33]. We found that to be true on average, but also that the risk of undetected heterogeneity is much higher when the number of studies to be meta-analysed is small. This reduced likelihood in identifying true underlying heterogeneity in small meta-analyses appears to be compensated for by the relatively larger positive bias in the bootstrap method, for those cases.

Even when heterogeneity is detected, it is not uncommon for researchers to ignore it and use a fixed-effect method. Such an approach has implications for the generalisability of the conclusions beyond the specific conditions and locations pertaining to the included studies. For full generalisability, a random-effects approach is required. The distributions of the between-study variance estimates from the various methods we used demonstrated that the bootstrap approach was better at detecting low heterogeneity levels in the real data, as in the simulations. Re-analysing all meta-analyses with this method we found that 19 to 21% of the statistical conclusions change, when heterogeneity is detected and ignored. The rates were much lower when the standard DerSimonian-Laird method did not detect heterogeneity, between approximately 1% (inverse variance) and 3% (Mantel-Haenszel), depending on the approach. When heterogeneity was detected and the random-effects model was used, we found that the statistical conclusions changed for approximately 2.5% of analyses, under the bootstrap.

Amongst the methods that permitted a zero-heterogeneity estimate, the bootstrapped DerSimonian-Laird performed best on the basis of our simulations – despite its higher bias for small meta-analyses. Even so, the high rate of failure to detect existing heterogeneity indicates that positive-only heterogeneity methods may be preferred, depending on the setting. MVa and BP, the Bayesian estimators proposed by Sidik and Jonkman [17] and Ruhkin [19] respectively, performed very well when two or three studies were meta-analysed and should be considered.

### Conclusions

Not surprisingly, detecting heterogeneity and accurately estimating it when the number of studies being meta-analysed is very small is difficult, if not impossible, yet more than half of all the meta-analyses in the Cochrane database include just two or three studies.

Current practice assumes that a zero between-study variance estimate leads to a more reliable meta-analysis, while high levels of estimated heterogeneity are alarming and potentially prohibitive for an analysis. However, our results indicate that estimates of zero (or even low) heterogeneity should also be a concern since heterogeneity is very likely present but undetected (or underestimated). Although the bootstrapped DerSimonian-Laird leads to a small improvement over the standard random-effects model, the problem largely remains, especially for very small meta-analyses. One possible solution in these cases is to arbitrarily assume moderate or large levels of heterogeneity and to test the sensitivity of the conclusions to these degrees of unobserved heterogeneity. This sensitivity-analysis approach, along with the bootstrapped DerSimonian-Laird have been included in an update for *metaan* [34]. Alternatively, confidence intervals for the bootstrapped DerSimonian-Laird estimate can easily be obtained and considered for sensitivity-analyses, but we found them to be of little value when the number of studies was small.

Finally, we must caution researchers against ignoring heterogeneity when detected. The narrower confidence intervals for the estimate and the fact that results are more likely to be statistically significant under the fixed-effect approach, should not influence their decision. Perhaps software developers should not offer a choice and sensitivity analyses should be reported as standard.

## Supporting Information

File S1 (DOCX)
