# Supplementary material for: A Re-Analysis of the Cochrane Library Data: The Dangers of Unobserved Heterogeneity in Meta-Analyses
Source: PLoS One. 2013 Jul 26;8(7):e69930. doi: 10.1371/journal.pone.0069930 (PMC3724681; doi:10.1371/journal.pone.0069930)
Supplement: File S1 — (DOCX) [file pone.0069930.s001.docx]

Figure S1: Between-study variance estimates by method type (main meta-analyses only)*

* truncated to 0.5 for better visualisation

Figure S2: Between-study variance estimates by meta-analysis size (main and subgroup)*^†^

* truncated to 0.5 for better visualisation

^†^ note that DLb identifies heterogeneity more often in all scenarios but some of the estimates are high (and not displayed in this graph)

Figure S3: Between-study variance estimates by number of studies, for the DerSimonian-Laird method and its non-parametric bootstrap*

*estimates have been constrained to the [0, 1] range, for better visualisation

Table S1: Mean bias (absolute mean bias)

| t^2=.01 | Normal | | | | | Skew normal (skewness=2, kurtosis=9) | | | | |
| --- | --- | --- | --- | --- | --- | --- | --- | --- | --- | --- |
| method | 2 | 3 | 5 | 10 | 20 | 2 | 3 | 5 | 10 | 20 |
| VC | 0.080  (0.094) | 0.064  (0.076) | 0.051  (0.063) | 0.036  (0.047) | 0.024  (0.034) | 0.084  (0.097) | 0.067  (0.079) | 0.050  (0.062) | 0.036  (0.047) | 0.025  (0.035) |
| DL | 0.080  (0.094) | 0.046  (0.058) | 0.024  (0.036) | 0.011  (0.021) | 0.005  (0.014) | 0.084  (0.097) | 0.047  (0.059) | 0.024  (0.035) | 0.011  (0.022) | 0.006  (0.015) |
| ML | 0.009  (0.026) | 0.008  (0.025) | 0.006  (0.021) | 0.003  (0.016) | 0.001  (0.012) | 0.010  (0.028) | 0.008  (0.025) | 0.005  (0.021) | 0.003  (0.016) | 0.001  (0.013) |
| REML | 0.081  (0.095) | 0.050  (0.063) | 0.026  (0.038) | 0.011  (0.021) | 0.004  (0.014) | 0.085  (0.098) | 0.051  (0.064) | 0.026  (0.038) | 0.011  (0.022) | 0.005  (0.014) |
| MVa | 0.085  (0.093) | 0.082  (0.086) | 0.080  (0.081) | 0.075  (0.075) | 0.072  (0.072) | 0.089  (0.096) | 0.085  (0.089) | 0.079  (0.081) | 0.075  (0.076) | 0.072  (0.072) |
| MVb | 0.082  (0.092) | 0.062  (0.070) | 0.047  (0.052) | 0.032  (0.035) | 0.021  (0.024) | 0.086  (0.095) | 0.065  (0.072) | 0.046  (0.052) | 0.032  (0.036) | 0.022  (0.025) |
| VC2 | 0.080  (0.094) | 0.061  (0.073) | 0.043  (0.054) | 0.025  (0.035) | 0.014  (0.022) | 0.084  (0.097) | 0.063  (0.075) | 0.042  (0.053) | 0.025  (0.035) | 0.015  (0.024) |
| DL2 | 0.080  (0.094) | 0.053  (0.065) | 0.032  (0.044) | 0.017  (0.027) | 0.008  (0.018) | 0.084  (0.097) | 0.055  (0.067) | 0.031  (0.043) | 0.017  (0.027) | 0.009  (0.018) |
| B0 | 0.020  (0.034) | 0.027  (0.040) | 0.030  (0.043) | 0.026  (0.037) | 0.016  (0.028) | 0.021  (0.035) | 0.028  (0.041) | 0.030  (0.042) | 0.026  (0.038) | 0.016  (0.028) |
| BP | 0.050  (0.055) | 0.080  (0.082) | 0.113  (0.113) | 0.140  (0.140) | 0.156  (0.156) | 0.052  (0.057) | 0.083  (0.085) | 0.112  (0.113) | 0.141  (0.141) | 0.156  (0.156) |
| DLi | 0.087  (0.087) | 0.052  (0.052) | 0.030  (0.030) | 0.016  (0.017) | 0.009  (0.011) | 0.090  (0.091) | 0.053  (0.054) | 0.029  (0.030) | 0.016  (0.017) | 0.010  (0.011) |
| DLb | 0.105  (0.118) | 0.069  (0.078) | 0.040  (0.047) | 0.019  (0.025) | 0.009  (0.015) | 0.110  (0.122) | 0.072  (0.081) | 0.039  (0.046) | 0.019  (0.025) | 0.010  (0.016) |
| t^2=.03 |  |  |  |  |  |  |  |  |  |  |
| method | 2 | 3 | 5 | 10 | 20 | 2 | 3 | 5 | 10 | 20 |
| VC | 0.081  (0.107) | 0.060  (0.085) | 0.047  (0.071) | 0.031  (0.053) | 0.020  (0.040) | 0.082  (0.109) | 0.063  (0.087) | 0.047  (0.070) | 0.032  (0.054) | 0.021  (0.041) |
| DL | 0.081  (0.107) | 0.043  (0.067) | 0.022  (0.044) | 0.009  (0.028) | 0.004  (0.019) | 0.082  (0.109) | 0.045  (0.069) | 0.022  (0.044) | 0.010  (0.029) | 0.004  (0.021) |
| ML | 0.002  (0.037) | 0.002  (0.035) | 0.001  (0.030) | -0.001  (0.023) | -0.001  (0.017) | 0.003  (0.038) | 0.003  (0.035) | 0.001  (0.031) | -0.000  (0.025) | -0.001  (0.019) |
| REML | 0.081  (0.108) | 0.048  (0.072) | 0.025  (0.047) | 0.010  (0.028) | 0.003  (0.019) | 0.083  (0.109) | 0.049  (0.074) | 0.025  (0.047) | 0.010  (0.030) | 0.003  (0.021) |
| MVa | 0.084  (0.102) | 0.079  (0.090) | 0.077  (0.082) | 0.073  (0.075) | 0.070  (0.070) | 0.086  (0.104) | 0.081  (0.092) | 0.077  (0.082) | 0.073  (0.075) | 0.071  (0.071) |
| MVb | 0.082  (0.105) | 0.059  (0.078) | 0.043  (0.060) | 0.028  (0.041) | 0.018  (0.030) | 0.084  (0.107) | 0.061  (0.081) | 0.043  (0.060) | 0.028  (0.042) | 0.019  (0.031) |
| VC2 | 0.081  (0.107) | 0.058  (0.081) | 0.042  (0.062) | 0.024  (0.041) | 0.013  (0.027) | 0.082  (0.109) | 0.061  (0.084) | 0.041  (0.062) | 0.024  (0.042) | 0.014  (0.029) |
| DL2 | 0.081  (0.107) | 0.050  (0.074) | 0.029  (0.051) | 0.014  (0.033) | 0.007  (0.023) | 0.082  (0.109) | 0.052  (0.076) | 0.029  (0.052) | 0.014  (0.034) | 0.007  (0.024) |
| B0 | 0.014  (0.042) | 0.020  (0.046) | 0.024  (0.049) | 0.020  (0.043) | 0.011  (0.034) | 0.014  (0.042) | 0.021  (0.047) | 0.024  (0.049) | 0.021  (0.044) | 0.012  (0.035) |
| BP | 0.044  (0.058) | 0.075  (0.081) | 0.109  (0.110) | 0.139  (0.139) | 0.155  (0.155) | 0.045  (0.059) | 0.077  (0.083) | 0.109  (0.110) | 0.138  (0.138) | 0.155  (0.155) |
| DLi | 0.087  (0.100) | 0.049  (0.061) | 0.027  (0.039) | 0.013  (0.024) | 0.007  (0.016) | 0.088  (0.102) | 0.051  (0.064) | 0.027  (0.039) | 0.014  (0.025) | 0.007  (0.018) |
| DLb | 0.108  (0.132) | 0.067  (0.086) | 0.038  (0.053) | 0.017  (0.030) | 0.008  (0.019) | 0.110  (0.134) | 0.070  (0.090) | 0.038  (0.054) | 0.017  (0.031) | 0.008  (0.020) |
| t^2=.1 |  |  |  |  |  |  |  |  |  |  |
| method | 2 | 3 | 5 | 10 | 20 | 2 | 3 | 5 | 10 | 20 |
| VC | 0.077  (0.144) | 0.054  (0.114) | 0.039  (0.094) | 0.023  (0.072) | 0.014  (0.054) | 0.077  (0.145) | 0.057  (0.120) | 0.041  (0.098) | 0.024  (0.075) | 0.015  (0.057) |
| DL | 0.077  (0.144) | 0.039  (0.097) | 0.019  (0.068) | 0.006  (0.045) | 0.002  (0.032) | 0.077  (0.145) | 0.041  (0.102) | 0.020  (0.074) | 0.007  (0.052) | 0.002  (0.039) |
| ML | -0.019  (0.069) | -0.015  (0.064) | -0.010  (0.056) | -0.008  (0.042) | -0.005  (0.030) | -0.019  (0.071) | -0.013  (0.068) | -0.009  (0.061) | -0.008  (0.049) | -0.006  (0.038) |
| REML | 0.078  (0.144) | 0.044  (0.102) | 0.022  (0.072) | 0.007  (0.047) | 0.002  (0.032) | 0.078  (0.146) | 0.047  (0.108) | 0.023  (0.078) | 0.006  (0.053) | 0.001  (0.039) |
| MVa | 0.079  (0.132) | 0.073  (0.110) | 0.071  (0.094) | 0.067  (0.078) | 0.066  (0.070) | 0.078  (0.133) | 0.075  (0.115) | 0.072  (0.098) | 0.067  (0.081) | 0.066  (0.072) |
| MVb | 0.079  (0.142) | 0.053  (0.108) | 0.037  (0.084) | 0.020  (0.060) | 0.011  (0.044) | 0.079  (0.144) | 0.056  (0.113) | 0.039  (0.089) | 0.021  (0.064) | 0.012  (0.048) |
| VC2 | 0.077  (0.144) | 0.055  (0.110) | 0.039  (0.084) | 0.022  (0.057) | 0.013  (0.039) | 0.077  (0.145) | 0.057  (0.116) | 0.040  (0.089) | 0.022  (0.063) | 0.013  (0.045) |
| DL2 | 0.077  (0.144) | 0.044  (0.103) | 0.024  (0.075) | 0.009  (0.051) | 0.004  (0.036) | 0.077  (0.145) | 0.047  (0.108) | 0.025  (0.080) | 0.009  (0.056) | 0.003  (0.041) |
| B0 | -0.009  (0.065) | 0.001  (0.067) | 0.008  (0.068) | 0.007  (0.060) | 0.001  (0.049) | -0.009  (0.067) | 0.002  (0.071) | 0.010  (0.071) | 0.008  (0.063) | 0.001  (0.051) |
| BP | 0.023  (0.073) | 0.059  (0.088) | 0.098  (0.110) | 0.132  (0.135) | 0.152  (0.152) | 0.023  (0.074) | 0.060  (0.091) | 0.098  (0.112) | 0.131  (0.135) | 0.152  (0.152) |
| DLi | 0.083  (0.138) | 0.044  (0.092) | 0.023  (0.064) | 0.009  (0.043) | 0.004  (0.030) | 0.083  (0.139) | 0.047  (0.097) | 0.024  (0.070) | 0.009  (0.050) | 0.004  (0.038) |
| DLb | 0.110  (0.171) | 0.068  (0.117) | 0.036  (0.076) | 0.014  (0.046) | 0.005  (0.031) | 0.110  (0.173) | 0.071  (0.123) | 0.038  (0.082) | 0.014  (0.053) | 0.006  (0.038) |

Table S2: Coverage (% of non-zero between-study variance estimates)

| t^2=.01 | Normal | | | | | Skew normal (skewness=2, kurtosis=9) | | | | |
| --- | --- | --- | --- | --- | --- | --- | --- | --- | --- | --- |
| method | 2 | 3 | 5 | 10 | 20 | 2 | 3 | 5 | 10 | 20 |
| VC | 94.3  (33.4) | 94.3  (37.3) | 93.9  (41.0) | 93.3  (45.5) | 93.6  (48.7) | 94.4  (33.5) | 94.3  (38.3) | 94.6  (40.9) | 94.2  (44.9) | 94.0  (48.7) |
| DL | 94.3  (33.4) | 94.3  (40.0) | 93.9  (47.1) | 93.6  (55.3) | 93.8  (63.1) | 94.4  (33.5) | 94.5  (41.0) | 94.8  (47.0) | 94.5  (54.6) | 94.4  (61.6) |
| ML | 92.7  (11.1) | 92.6  (16.7) | 92.3  (24.6) | 92.0  (38.7) | 93.0  (54.1) | 92.7  (10.8) | 92.7  (16.9) | 93.2  (24.1) | 93.2  (36.4) | 93.4  (52.5) |
| REML | 94.3  (33.8) | 94.2  (39.0) | 93.8  (44.8) | 93.4  (54.2) | 93.9  (64.3) | 94.5  (33.9) | 94.3  (39.8) | 94.7  (44.5) | 94.2  (52.1) | 94.2  (61.9) |
| MVa | 95.6  (100.0) | 96.6  (100.0) | 97.8  (100.0) | 98.6  (100.0) | 98.9  (100.0) | 95.7  (100.0) | 96.7  (100.0) | 98.0  (100.0) | 98.7  (100.0) | 98.9  (100.0) |
| MVb | 95.0  (100.0) | 95.2  (100.0) | 95.4  (100.0) | 95.3  (100.0) | 95.7  (100.0) | 94.9  (100.0) | 95.4  (100.0) | 95.9  (100.0) | 96.1  (100.0) | 96.1  (100.0) |
| VC2 | 94.3  (33.4) | 94.6  (41.4) | 94.3  (49.3) | 94.1  (57.4) | 94.3  (64.6) | 94.4  (33.5) | 94.6  (42.1) | 95.1  (48.8) | 94.9  (57.1) | 94.7  (63.0) |
| DL2 | 94.3  (33.4) | 94.3  (40.0) | 93.8  (46.5) | 93.5  (54.5) | 93.8  (62.4) | 94.4  (33.5) | 94.4  (41.0) | 94.7  (46.4) | 94.4  (53.7) | 94.2  (61.0) |
| B0 | 93.8  (33.5) | 94.0  (37.3) | 93.7  (40.6) | 92.9  (43.8) | 93.0  (42.5) | 94.0  (33.6) | 94.0  (38.3) | 94.4  (40.5) | 94.0  (43.3) | 93.4  (42.7) |
| BP | 95.9  (100.0) | 97.5  (100.0) | 98.9  (100.0) | 99.5  (100.0) | 99.8  (100.0) | 96.0  (100.0) | 97.6  (100.0) | 98.9  (100.0) | 99.5  (100.0) | 99.7  (100.0) |
| DLi | 96.3  (100.0) | 96.1  (100.0) | 96.2  (100.0) | 95.7  (100.0) | 95.5  (100.0) | 96.2  (100.0) | 96.5  (100.0) | 96.5  (100.0) | 96.2  (100.0) | 95.9  (100.0) |
| DLb | 94.8  (42.1) | 95.2  (62.3) | 95.2  (84.1) | 95.0  (95.6) | 95.0  (98.9) | 94.9  (42.7) | 95.4  (63.1) | 96.0  (83.1) | 95.6  (95.6) | 95.3  (98.8) |
| PL | 98.2  (11.1) | 97.2  (16.7) | 96.1  (24.6) | 95.2  (38.7) | 94.9  (54.1) | 98.5  (10.8) | 97.3  (16.9) | 96.6  (24.1) | 95.7  (36.4) | 95.3  (52.5) |
| t^2=.03 |  |  |  |  |  |  |  |  |  |  |
| method | 2 | 3 | 5 | 10 | 20 | 2 | 3 | 5 | 10 | 20 |
| VC | 92.8  (35.7) | 92.5  (39.8) | 92.0  (44.0) | 91.5  (49.4) | 91.5  (54.3) | 93.1  (35.0) | 93.0  (40.0) | 92.8  (43.7) | 92.6  (48.5) | 92.1  (54.1) |
| DL | 92.8  (35.7) | 92.7  (43.0) | 92.7  (52.0) | 92.6  (62.6) | 92.9  (72.9) | 93.1  (35.0) | 93.3  (43.1) | 93.5  (50.8) | 93.5  (60.7) | 93.5  (70.8) |
| ML | 90.6  (12.8) | 90.3  (19.6) | 90.5  (30.5) | 90.6  (47.4) | 91.8  (65.9) | 90.8  (12.4) | 91.0  (19.3) | 91.5  (28.4) | 91.8  (44.0) | 92.3  (62.2) |
| REML | 92.9  (36.1) | 92.5  (42.0) | 92.6  (50.2) | 92.5  (62.0) | 92.9  (74.1) | 93.1  (35.4) | 93.1  (41.9) | 93.4  (48.6) | 93.1  (58.9) | 93.3  (70.6) |
| MVa | 94.4  (100.0) | 95.6  (100.0) | 97.0  (100.0) | 98.0  (100.0) | 98.6  (100.0) | 94.7  (100.0) | 95.9  (100.0) | 97.5  (100.0) | 98.2  (100.0) | 98.6  (100.0) |
| MVb | 93.6  (100.0) | 93.7  (100.0) | 94.1  (100.0) | 94.1  (100.0) | 94.3  (100.0) | 93.7  (100.0) | 94.3  (100.0) | 94.7  (100.0) | 94.9  (100.0) | 94.8  (100.0) |
| VC2 | 92.8  (35.7) | 93.0  (44.3) | 93.2  (53.9) | 93.1  (64.2) | 93.4  (73.9) | 93.1  (35.0) | 93.5  (44.2) | 93.8  (52.5) | 94.0  (62.6) | 93.9  (71.8) |
| DL2 | 92.8  (35.7) | 92.6  (43.0) | 92.5  (51.2) | 92.3  (61.6) | 92.7  (72.1) | 93.1  (35.0) | 93.2  (43.1) | 93.3  (50.2) | 93.3  (59.6) | 93.2  (70.1) |
| B0 | 92.2  (35.8) | 92.0  (39.8) | 91.6  (43.6) | 91.0  (47.6) | 90.6  (48.0) | 92.4  (35.1) | 92.5  (40.0) | 92.5  (43.3) | 92.2  (46.9) | 91.3  (47.8) |
| BP | 94.9  (100.0) | 96.7  (100.0) | 98.4  (100.0) | 99.3  (100.0) | 99.6  (100.0) | 94.9  (100.0) | 96.9  (100.0) | 98.6  (100.0) | 99.4  (100.0) | 99.6  (100.0) |
| DLi | 95.1  (100.0) | 94.7  (100.0) | 95.0  (100.0) | 94.5  (100.0) | 94.4  (100.0) | 95.1  (100.0) | 95.5  (100.0) | 95.5  (100.0) | 95.3  (100.0) | 95.0  (100.0) |
| DLb | 93.6  (44.3) | 93.8  (64.8) | 94.3  (86.0) | 94.2  (96.7) | 94.1  (99.4) | 93.7  (43.9) | 94.4  (64.4) | 95.0  (84.9) | 94.7  (96.3) | 94.5  (99.2) |
| PL | 97.6  (12.8) | 96.2  (19.6) | 95.3  (30.5) | 94.4  (47.4) | 94.2  (65.9) | 97.9  (12.4) | 96.5  (19.3) | 95.8  (28.4) | 94.9  (44.0) | 94.5  (62.2) |
| t^2=.1 |  |  |  |  |  |  |  |  |  |  |
| method | 2 | 3 | 5 | 10 | 20 | 2 | 3 | 5 | 10 | 20 |
| VC | 89.7  (40.2) | 89.4  (46.2) | 89.0  (52.4) | 89.0  (60.1) | 89.8  (67.9) | 90.5  (38.5) | 90.2  (44.8) | 90.0  (50.7) | 89.9  (57.8) | 90.2  (66.5) |
| DL | 89.7  (40.2) | 89.9  (50.2) | 90.4  (61.8) | 91.3  (74.8) | 92.5  (84.8) | 90.5  (38.5) | 90.8  (48.5) | 90.9  (58.9) | 91.6  (71.4) | 92.4  (82.3) |
| ML | 86.0  (16.7) | 86.0  (27.0) | 87.4  (42.2) | 89.0  (62.7) | 91.4  (80.0) | 87.1  (15.8) | 87.3  (25.6) | 88.0  (38.3) | 89.4  (57.4) | 90.9  (75.9) |
| REML | 89.8  (41.0) | 89.7  (49.4) | 90.3  (60.3) | 91.2  (74.1) | 92.6  (85.3) | 90.6  (39.5) | 90.6  (48.0) | 90.8  (57.1) | 91.2  (69.9) | 92.1  (81.9) |
| MVa | 91.6  (100.0) | 93.3  (100.0) | 95.2  (100.0) | 96.7  (100.0) | 97.7  (100.0) | 92.3  (100.0) | 93.9  (100.0) | 95.9  (100.0) | 97.1  (100.0) | 97.7  (100.0) |
| MVb | 90.4  (100.0) | 90.9  (100.0) | 91.4  (100.0) | 91.8  (100.0) | 92.5  (100.0) | 91.2  (100.0) | 91.8  (100.0) | 92.2  (100.0) | 92.7  (100.0) | 92.9  (100.0) |
| VC2 | 89.7  (40.2) | 90.4  (51.3) | 91.1  (63.3) | 92.0  (75.8) | 93.2  (85.4) | 90.5  (38.5) | 91.1  (49.5) | 91.6  (60.4) | 92.3  (72.8) | 93.0  (82.9) |
| DL2 | 89.7  (40.2) | 89.8  (50.2) | 90.1  (61.0) | 90.7  (73.7) | 92.1  (84.2) | 90.5  (38.5) | 90.6  (48.5) | 90.6  (58.3) | 91.2  (70.3) | 92.1  (81.7) |
| B0 | 88.3  (40.2) | 88.3  (46.2) | 88.3  (52.0) | 88.1  (58.4) | 88.3  (62.5) | 89.1  (38.6) | 89.2  (44.8) | 89.2  (50.3) | 89.1  (56.2) | 88.8  (60.9) |
| BP | 91.8  (100.0) | 94.5  (100.0) | 97.0  (100.0) | 98.5  (100.0) | 99.2  (100.0) | 92.5  (100.0) | 95.0  (100.0) | 97.3  (100.0) | 98.7  (100.0) | 99.2  (100.0) |
| DLi | 92.1  (100.0) | 91.9  (100.0) | 92.4  (100.0) | 92.7  (100.0) | 93.4  (100.0) | 92.8  (100.0) | 93.2  (100.0) | 93.0  (100.0) | 93.1  (100.0) | 93.4  (100.0) |
| DLb | 90.7  (48.5) | 91.5  (69.6) | 92.3  (89.3) | 92.8  (98.0) | 93.5  (99.7) | 91.4  (47.1) | 92.3  (68.3) | 92.8  (88.1) | 93.0  (97.5) | 93.4  (99.6) |
| PL | 95.6  (16.7) | 94.1  (27.0) | 93.4  (42.2) | 93.2  (62.7) | 93.9  (80.0) | 96.2  (15.8) | 94.8  (25.6) | 93.8  (38.3) | 93.3  (57.4) | 93.4  (75.9) |

Table S3: Median error interval estimation (25^th^ and 75^th^ centiles)

| t^2=.01 | Normal | | | | | Skew normal (skewness=2, kurtosis=9) | | | | |
| --- | --- | --- | --- | --- | --- | --- | --- | --- | --- | --- |
| method | 2 | 3 | 5 | 10 | 20 | 2 | 3 | 5 | 10 | 20 |
| VC | 0.96  (0.90-1.21) | 0.96  (0.89-1.29) | 0.94  (0.87-1.37) | 0.93  (0.85-1.39) | 0.91  (0.85-1.33) | 0.96  (0.90-1.22) | 0.96  (0.89-1.32) | 0.94  (0.87-1.35) | 0.93  (0.85-1.37) | 0.91  (0.85-1.34) |
| DL | 0.96  (0.90-1.21) | 0.96  (0.89-1.25) | 0.95  (0.88-1.24) | 0.95  (0.87-1.20) | 0.97  (0.87-1.15) | 0.96  (0.90-1.22) | 0.96  (0.89-1.28) | 0.95  (0.88-1.24) | 0.95  (0.87-1.19) | 0.96  (0.87-1.15) |
| ML | 0.94  (0.87-0.97) | 0.93  (0.86-0.97) | 0.91  (0.85-0.97) | 0.91  (0.85-1.05) | 0.91  (0.86-1.07) | 0.94  (0.87-0.97) | 0.93  (0.85-0.97) | 0.91  (0.85-0.97) | 0.90  (0.85-1.03) | 0.91  (0.85-1.07) |
| REML | 0.96  (0.90-1.22) | 0.96  (0.89-1.24) | 0.95  (0.87-1.21) | 0.94  (0.87-1.17) | 0.96  (0.87-1.12) | 0.97  (0.90-1.22) | 0.96  (0.89-1.25) | 0.95  (0.87-1.20) | 0.93  (0.86-1.15) | 0.95  (0.86-1.12) |
| MVa | 1.01  (0.95-1.33) | 1.14  (0.98-1.49) | 1.28  (1.08-1.60) | 1.39  (1.21-1.64) | 1.46  (1.31-1.63) | 1.02  (0.95-1.34) | 1.15  (0.98-1.51) | 1.28  (1.07-1.59) | 1.39  (1.21-1.64) | 1.45  (1.31-1.63) |
| MVb | 0.98  (0.93-1.21) | 0.98  (0.94-1.29) | 0.99  (0.95-1.33) | 1.00  (0.96-1.34) | 1.00  (0.97-1.28) | 0.98  (0.93-1.22) | 0.98  (0.94-1.30) | 0.99  (0.95-1.32) | 1.00  (0.96-1.32) | 1.01  (0.97-1.29) |
| VC2 | 0.96  (0.90-1.21) | 0.96  (0.89-1.33) | 0.96  (0.88-1.36) | 0.98  (0.87-1.30) | 1.00  (0.87-1.23) | 0.96  (0.90-1.22) | 0.96  (0.89-1.35) | 0.96  (0.88-1.34) | 0.97  (0.87-1.29) | 1.00  (0.87-1.23) |
| DL2 | 0.96  (0.90-1.21) | 0.96  (0.89-1.25) | 0.95  (0.87-1.25) | 0.94  (0.87-1.22) | 0.96  (0.86-1.17) | 0.96  (0.90-1.22) | 0.96  (0.89-1.27) | 0.95  (0.87-1.25) | 0.94  (0.86-1.21) | 0.95  (0.86-1.17) |
| B0 | 0.96  (0.90-1.03) | 0.95  (0.88-1.12) | 0.94  (0.87-1.24) | 0.92  (0.85-1.30) | 0.89  (0.85-1.23) | 0.96  (0.90-1.04) | 0.96  (0.88-1.15) | 0.94  (0.87-1.23) | 0.92  (0.85-1.28) | 0.89  (0.85-1.23) |
| BP | 1.05  (0.97-1.27) | 1.23  (1.06-1.52) | 1.47  (1.25-1.78) | 1.69  (1.48-1.96) | 1.83  (1.66-2.03) | 1.06  (0.97-1.28) | 1.24  (1.06-1.54) | 1.46  (1.24-1.77) | 1.70  (1.48-1.96) | 1.83  (1.65-2.03) |
| DLi | 1.00  (1.00-1.21) | 1.00  (1.00-1.25) | 1.00  (1.00-1.24) | 1.00  (1.00-1.20) | 1.00  (1.00-1.15) | 1.00  (1.00-1.22) | 1.00  (1.00-1.28) | 1.00  (1.00-1.24) | 1.00  (1.00-1.19) | 1.00  (1.00-1.15) |
| DLb | 0.97  (0.92-1.36) | 0.99  (0.92-1.43) | 1.05  (0.92-1.37) | 1.05  (0.92-1.26) | 1.03  (0.92-1.18) | 0.97  (0.92-1.37) | 0.99  (0.92-1.45) | 1.05  (0.92-1.37) | 1.05  (0.92-1.26) | 1.03  (0.92-1.18) |
| PL | 1.27  (1.16-1.52) | 1.16  (1.05-1.44) | 1.09  (0.98-1.36) | 1.05  (0.93-1.28) | 1.03  (0.92-1.19) | 1.28  (1.16-1.53) | 1.17  (1.05-1.46) | 1.09  (0.98-1.35) | 1.04  (0.93-1.26) | 1.02  (0.91-1.19) |
| t^2=.03 |  |  |  |  |  |  |  |  |  |  |
| method | 2 | 3 | 5 | 10 | 20 | 2 | 3 | 5 | 10 | 20 |
| VC | 0.94  (0.85-1.21) | 0.93  (0.82-1.27) | 0.91  (0.80-1.33) | 0.90  (0.79-1.33) | 0.91  (0.79-1.29) | 0.94  (0.84-1.20) | 0.93  (0.82-1.29) | 0.91  (0.80-1.32) | 0.89  (0.79-1.32) | 0.91  (0.79-1.29) |
| DL | 0.94  (0.85-1.21) | 0.93  (0.83-1.23) | 0.93  (0.82-1.22) | 0.95  (0.82-1.18) | 0.97  (0.84-1.13) | 0.94  (0.84-1.20) | 0.93  (0.83-1.25) | 0.92  (0.82-1.21) | 0.93  (0.81-1.16) | 0.95  (0.83-1.13) |
| ML | 0.90  (0.80-0.95) | 0.88  (0.78-0.95) | 0.87  (0.78-0.97) | 0.88  (0.79-1.05) | 0.92  (0.82-1.07) | 0.90  (0.80-0.95) | 0.88  (0.78-0.95) | 0.87  (0.78-0.96) | 0.87  (0.79-1.04) | 0.90  (0.80-1.06) |
| REML | 0.94  (0.85-1.22) | 0.93  (0.83-1.22) | 0.92  (0.82-1.20) | 0.94  (0.82-1.16) | 0.96  (0.84-1.12) | 0.94  (0.84-1.21) | 0.93  (0.83-1.23) | 0.92  (0.81-1.19) | 0.92  (0.81-1.14) | 0.94  (0.83-1.11) |
| MVa | 0.98  (0.90-1.31) | 1.09  (0.93-1.43) | 1.22  (1.02-1.52) | 1.32  (1.15-1.54) | 1.38  (1.24-1.53) | 0.99  (0.90-1.29) | 1.10  (0.93-1.44) | 1.22  (1.02-1.51) | 1.32  (1.14-1.54) | 1.37  (1.24-1.54) |
| MVb | 0.95  (0.87-1.21) | 0.95  (0.87-1.26) | 0.95  (0.88-1.30) | 0.95  (0.89-1.29) | 0.96  (0.90-1.24) | 0.95  (0.87-1.20) | 0.95  (0.87-1.27) | 0.95  (0.88-1.29) | 0.95  (0.89-1.28) | 0.96  (0.90-1.25) |
| VC2 | 0.94  (0.85-1.21) | 0.94  (0.84-1.31) | 0.95  (0.83-1.33) | 0.98  (0.83-1.27) | 1.00  (0.85-1.21) | 0.94  (0.84-1.20) | 0.94  (0.84-1.32) | 0.94  (0.83-1.31) | 0.96  (0.82-1.27) | 1.00  (0.84-1.21) |
| DL2 | 0.94  (0.85-1.21) | 0.93  (0.83-1.23) | 0.92  (0.82-1.23) | 0.93  (0.82-1.19) | 0.96  (0.83-1.15) | 0.94  (0.84-1.20) | 0.93  (0.83-1.24) | 0.92  (0.81-1.22) | 0.92  (0.81-1.19) | 0.95  (0.82-1.15) |
| B0 | 0.93  (0.84-1.01) | 0.92  (0.82-1.09) | 0.90  (0.80-1.19) | 0.88  (0.79-1.24) | 0.86  (0.78-1.19) | 0.93  (0.84-1.01) | 0.92  (0.82-1.11) | 0.90  (0.80-1.19) | 0.88  (0.78-1.24) | 0.86  (0.78-1.19) |
| BP | 1.02  (0.92-1.23) | 1.18  (1.01-1.45) | 1.39  (1.18-1.67) | 1.59  (1.39-1.83) | 1.71  (1.55-1.89) | 1.02  (0.92-1.23) | 1.18  (1.01-1.46) | 1.38  (1.17-1.67) | 1.59  (1.39-1.83) | 1.71  (1.55-1.89) |
| DLi | 0.97  (0.93-1.21) | 0.97  (0.93-1.23) | 0.97  (0.92-1.22) | 0.97  (0.92-1.18) | 0.99  (0.93-1.13) | 0.97  (0.93-1.20) | 0.97  (0.92-1.25) | 0.96  (0.92-1.21) | 0.96  (0.92-1.16) | 0.97  (0.92-1.13) |
| DLb | 0.95  (0.87-1.35) | 0.97  (0.87-1.39) | 1.02  (0.88-1.33) | 1.03  (0.88-1.23) | 1.01  (0.89-1.15) | 0.95  (0.87-1.33) | 0.97  (0.87-1.40) | 1.01  (0.87-1.32) | 1.01  (0.87-1.22) | 1.00  (0.88-1.15) |
| PL | 1.21  (1.11-1.48) | 1.11  (0.99-1.40) | 1.05  (0.93-1.34) | 1.03  (0.89-1.26) | 1.03  (0.90-1.18) | 1.21  (1.11-1.48) | 1.11  (0.99-1.41) | 1.04  (0.92-1.32) | 1.01  (0.88-1.25) | 1.01  (0.88-1.18) |
| t^2=.1 |  |  |  |  |  |  |  |  |  |  |
| method | 2 | 3 | 5 | 10 | 20 | 2 | 3 | 5 | 10 | 20 |
| VC | 0.88  (0.75-1.20) | 0.88  (0.73-1.25) | 0.88  (0.71-1.28) | 0.90  (0.71-1.26) | 0.93  (0.75-1.22) | 0.88  (0.74-1.17) | 0.87  (0.72-1.24) | 0.86  (0.71-1.27) | 0.88  (0.70-1.26) | 0.92  (0.73-1.23) |
| DL | 0.88  (0.75-1.20) | 0.89  (0.75-1.21) | 0.91  (0.75-1.20) | 0.94  (0.79-1.15) | 0.97  (0.84-1.11) | 0.88  (0.74-1.17) | 0.88  (0.73-1.21) | 0.88  (0.73-1.18) | 0.91  (0.75-1.13) | 0.94  (0.80-1.11) |
| ML | 0.81  (0.68-0.91) | 0.81  (0.68-0.92) | 0.82  (0.69-0.99) | 0.88  (0.74-1.06) | 0.93  (0.81-1.07) | 0.81  (0.68-0.91) | 0.80  (0.67-0.91) | 0.80  (0.68-0.97) | 0.84  (0.71-1.04) | 0.90  (0.77-1.06) |
| REML | 0.88  (0.75-1.21) | 0.88  (0.74-1.21) | 0.91  (0.75-1.20) | 0.94  (0.79-1.15) | 0.97  (0.84-1.11) | 0.88  (0.74-1.17) | 0.87  (0.73-1.20) | 0.87  (0.73-1.17) | 0.90  (0.75-1.13) | 0.94  (0.80-1.11) |
| MVa | 0.93  (0.80-1.25) | 1.03  (0.85-1.35) | 1.14  (0.94-1.41) | 1.22  (1.06-1.42) | 1.26  (1.14-1.40) | 0.92  (0.79-1.22) | 1.01  (0.84-1.35) | 1.12  (0.92-1.40) | 1.20  (1.03-1.41) | 1.25  (1.12-1.41) |
| MVb | 0.89  (0.77-1.20) | 0.89  (0.77-1.25) | 0.91  (0.78-1.26) | 0.94  (0.80-1.23) | 0.96  (0.83-1.19) | 0.88  (0.76-1.17) | 0.88  (0.77-1.23) | 0.89  (0.77-1.24) | 0.92  (0.79-1.22) | 0.95  (0.81-1.20) |
| VC2 | 0.88  (0.75-1.20) | 0.90  (0.75-1.28) | 0.94  (0.77-1.29) | 0.99  (0.81-1.23) | 1.01  (0.86-1.17) | 0.88  (0.74-1.17) | 0.89  (0.74-1.27) | 0.91  (0.74-1.27) | 0.95  (0.77-1.23) | 0.99  (0.82-1.18) |
| DL2 | 0.88  (0.75-1.20) | 0.88  (0.74-1.21) | 0.90  (0.74-1.20) | 0.93  (0.77-1.17) | 0.96  (0.83-1.13) | 0.88  (0.74-1.17) | 0.87  (0.73-1.20) | 0.87  (0.73-1.19) | 0.90  (0.74-1.15) | 0.94  (0.79-1.13) |
| B0 | 0.86  (0.73-0.97) | 0.86  (0.72-1.05) | 0.85  (0.71-1.14) | 0.87  (0.70-1.18) | 0.87  (0.72-1.14) | 0.85  (0.72-0.96) | 0.85  (0.71-1.05) | 0.84  (0.70-1.13) | 0.85  (0.70-1.17) | 0.87  (0.70-1.15) |
| BP | 0.94  (0.82-1.14) | 1.08  (0.91-1.33) | 1.26  (1.07-1.51) | 1.43  (1.26-1.64) | 1.53  (1.39-1.69) | 0.94  (0.81-1.13) | 1.08  (0.90-1.33) | 1.25  (1.05-1.51) | 1.42  (1.24-1.64) | 1.53  (1.38-1.69) |
| DLi | 0.91  (0.81-1.20) | 0.91  (0.82-1.21) | 0.93  (0.82-1.20) | 0.96  (0.84-1.15) | 0.98  (0.89-1.11) | 0.90  (0.81-1.17) | 0.90  (0.81-1.21) | 0.91  (0.81-1.18) | 0.92  (0.82-1.13) | 0.95  (0.85-1.11) |
| DLb | 0.90  (0.77-1.32) | 0.94  (0.79-1.35) | 0.99  (0.81-1.29) | 1.00  (0.84-1.19) | 1.00  (0.87-1.13) | 0.90  (0.76-1.29) | 0.92  (0.78-1.34) | 0.96  (0.79-1.27) | 0.97  (0.81-1.17) | 0.97  (0.84-1.12) |
| PL | 1.12  (0.98-1.42) | 1.04  (0.89-1.38) | 1.02  (0.85-1.33) | 1.02  (0.85-1.24) | 1.02  (0.89-1.16) | 1.11  (0.97-1.39) | 1.02  (0.88-1.37) | 0.99  (0.83-1.30) | 0.98  (0.81-1.22) | 0.99  (0.84-1.16) |

Table S4: Variation in terms of statistical conclusion between the standard approach and DLb for inverse variance meta-analyses, by number of studies

|  |  | RevMan method | |  |  |  |  |
| --- | --- | --- | --- | --- | --- | --- | --- |
|  |  | Fixed-effect ($\hat{\tau}_{DL}^{2}=0$) | | Fixed-effect ($\hat{\tau}_{DL}^{2}>0$) | | Random-effects DL ($\hat{\tau}_{DL}^{2}>0$) | |
|  |  | Counts (cell percentages) | | Counts (cell percentages) | | Counts (cell percentages) | |
| # of studies |  | NS | Sig | NS | Sig | NS | Sig |
| 2 | NS | 2341(56.2%)* | 28(0.7%)^†^ | 775(51.4%) | 349(23.2%) | 1009(68.8%) | 30(2.0%) |
|  | Sig | 0(0.0%)^‡^ | 1797(43.1%)^§^ | 0(0.0%) | 383(25.4%) | 0(0.0%) | 428(29.2%) |
| 3 | NS | 1007(52.1%) | 23(1.2%) | 377(37.9%) | 238(23.9%) | 605(57.2%) | 35(3.3%) |
|  | Sig | 0(0.0%) | 901(46.7%) | 0(0.0%) | 379(38.1%) | 1(0.1%) | 416(39.4%) |
| 4 | NS | 480(45.1%) | 12(1.1%) | 213(34.9%) | 108(17.7%) | 354(46.6%) | 27(3.6%) |
|  | Sig | 0(0.0%) | 572(53.8%) | 0(0.0%) | 289(47.4%) | 1(0.1%) | 378(49.7%) |
| 5 | NS | 225(40.7%) | 9(1.6%) | 129(31.5%) | 71(17.4%) | 256(45.1%) | 18(3.2%) |
|  | Sig | 0(0.0%) | 319(57.7%) | 1(0.2%) | 208(50.9%) | 1(0.2%) | 293(51.6%) |
| 6-9 | NS | 331(38.8%) | 10(1.2%) | 180(21.4%) | 116(13.8%) | 380(32.6%) | 23(2.0%) |
|  | Sig | 0(0.0%) | 511(60.0%) | 1(0.1%) | 546(64.8%) | 2(0.2%) | 762(65.3%) |
| 10+ | NS | 110(30.1%) | 2(0.5%) | 63(11.0%) | 54(9.4%) | 173(17.6%) | 7(0.7%) |
|  | Sig | 0(0.0%) | 254(69.4%) | 4(0.7%) | 453(78.9%) | 1(0.1%) | 804(81.6%) |

* Agreement in statistical conclusion (non-significant effect) between RevMan and DLb method

† Non agreement in statistical conclusion between RevMan (significant effect) and DLb method (non-significant effect)

‡ Non agreement in statistical conclusion between RevMan (significant effect) and DLb methods (non-significant effect)

§ Agreement in statistical conclusion (significant effect) between RevMan and DLb methods

Table S5: Variation in terms of statistical conclusion between the standard approach and DLb for Mantel-Haenszel meta-analyses, by number of studies

|  |  | RevMan method | |  |  |  |  |
| --- | --- | --- | --- | --- | --- | --- | --- |
|  |  | Fixed-effect ($\hat{\tau}_{DL}^{2}=0$) | | Fixed-effect ($\hat{\tau}_{DL}^{2}>0$) | | Random-effects DL ($\hat{\tau}_{DL}^{2}>0$) | |
|  |  | Counts (cell percentages) | | Counts (cell percentages) | | Counts (cell percentages) | |
| # of studies |  | NS | Sig | NS | Sig | NS | Sig |
| 2 | NS | 3760(74.6%) | 79(1.6%) | 1564(66.8%) | 504(21.5%) | 2850(76.8%) | 33(0.9%) |
|  | Sig | 21(0.4%) | 1181(23.4%) | 0(0.0%) | 245(10.5%) | 1(0.0%) | 814(21.9%) |
|  | No comp[**†**](http://en.wikipedia.org/wiki/Dagger_(typography)) | 0(0.0%) | 0(0.0%) | 27(1.2%) | 3(0.1%) | 11(0.3%) | 0(0.0%) |
| 3 | NS | 1797(71.3%) | 61(2.4%) | 842(57.8%) | 341(23.4%) | 1713(73.6%) | 48(2.1%) |
|  | Sig | 9(0.4%) | 653(25.9%) | 0(0.0%) | 269(18.5%) | 11(0.5%) | 553(23.7%) |
|  | No comp[**†**](http://en.wikipedia.org/wiki/Dagger_(typography)) | 0(0.0%) | 0(0.0%) | 4(0.3%) | 0(0.0%) | 4(0.2%) | 0(0.0%) |
| 4 | NS | 1040(69.1%) | 48(3.2%) | 522(51.9%) | 227(22.6%) | 1010(66.0%) | 56(3.7%) |
|  | Sig | 4(0.3%) | 414(27.5%) | 0(0.0%) | 253(25.1%) | 6(0.4%) | 458(29.9%) |
|  | No comp[**†**](http://en.wikipedia.org/wiki/Dagger_(typography)) | 0(0.0%) | 0(0.0%) | 4(0.4%) | 0(0.0%) | 0(0.0%) | 0(0.0%) |
| 5 | NS | 662(68.2%) | 36(3.7%) | 358(49.7%) | 141(19.6%) | 663(63.2%) | 30(2.9%) |
|  | Sig | 7(0.7%) | 266(27.4%) | 1(0.1%) | 219(30.4%) | 9(0.9%) | 346(33.0%) |
|  | No comp[**†**](http://en.wikipedia.org/wiki/Dagger_(typography)) | 0(0.0%) | 0(0.0%) | 1(0.1%) | 0(0.0%) | 1(0.1%) | 0(0.0%) |
| 6-9 | NS | 1010(65.0%) | 64(4.1%) | 619(43.3%) | 271(18.9%) | 1105(56.3%) | 63(3.2%) |
|  | Sig | 4(0.3%) | 476(30.6%) | 0(0.0%) | 540(37.7%) | 14(0.7%) | 780(39.8%) |
|  | No comp[**†**](http://en.wikipedia.org/wiki/Dagger_(typography)) | 0(0.0%) | 0(0.0%) | 1(0.1%) | 0(0.0%) | 0(0.0%) | 0(0.0%) |
| 10+ | NS | 621(60.5%) | 48(4.7%) | 401(28.9%) | 192(13.8%) | 682(41.5%) | 35(2.1%) |
|  | Sig | 5(0.5%) | 352(34.3%) | 12(0.9%) | 783(56.4%) | 24(1.5%) | 902(54.9%) |
|  | No comp[**†**](http://en.wikipedia.org/wiki/Dagger_(typography)) | 0(0.0%) | 0(0.0%) | 0(0.0%) | 0(0.0%) | 0(0.0%) | 0(0.0%) |

* Due to the RevMan issue with the calculation of study effects that were incomputable, for odds and risk ratios, some meta-analyses were not computable (when the remaining number of eligible studies was one or zero).

[†](http://en.wikipedia.org/wiki/Dagger_(typography)) Not computable (see above).

 Table S6: Variation in terms of statistical conclusion between the standard approach and DLb for Peto fixed-effect meta-analyses, by number of studies

|  |  | RevMan method | |  |  |
| --- | --- | --- | --- | --- | --- |
|  |  | Fixed-effect ($\hat{\tau}_{DL}^{2}=0$) | | Fixed-effect ($\hat{\tau}_{DL}^{2}>0$) | |
|  |  | Counts (cell percentages) | | Counts (cell percentages) | |
| # of studies |  | NS | Sig | NS | Sig |
| 2 | NS | 524(66.8%) | 6(0.8%) | 345(72.6%) | 82(17.3%) |
|  | Sig | 4(0.5%) | 251(32.0%) | 0(0.0%) | 48(10.1%) |
| 3 | NS | 268(67.2%) | 7(1.8%) | 230(62.3%) | 68(18.4%) |
|  | Sig | 0(0.0%) | 124(31.1%) | 0(0.0%) | 71(19.2%) |
| 4 | NS | 174(59.6%) | 7(2.4%) | 142(54.0%) | 59(22.4%) |
|  | Sig | 0(0.0%) | 111(38.0%) | 0(0.0%) | 62(23.6%) |
| 5 | NS | 99(53.8%) | 2(1.1%) | 127(56.7%) | 48(21.4%) |
|  | Sig | 0(0.0%) | 83(45.1%) | 0(0.0%) | 49(21.9%) |
| 6-9 | NS | 191(55.2%) | 7(2.0%) | 196(50.0%) | 40(10.2%) |
|  | Sig | 0(0.0%) | 148(42.8%) | 1(0.3%) | 155(39.5%) |
| 10+ | NS | 111(55.5%) | 7(3.5%) | 137(33.3%) | 62(15.1%) |
|  | Sig | 0(0.0%) | 82(41.0%) | 6(1.5%) | 206(50.1%) |
